# AMDA: an R package for the automated microarray data analysis

**DOI:** 10.1186/1471-2105-7-335

**Published:** 2006-07-06

**Authors:** Mattia Pelizzola, Norman Pavelka, Maria Foti, Paola Ricciardi-Castagnoli

**Affiliations:** 1 Department of Biotechnology and Biosciences, University of Milano-Bicocca, Piazza della Scienza 2, 20126 Milan, Italy

## Abstract

**Background:**

Microarrays are routinely used to assess mRNA transcript levels on a genome-wide scale. Large amount of microarray datasets are now available in several databases, and new experiments are constantly being performed. In spite of this fact, few and limited tools exist for quickly and easily analyzing the results. Microarray analysis can be challenging for researchers without the necessary training and it can be time-consuming for service providers with many users.

**Results:**

To address these problems we have developed an automated microarray data analysis (AMDA) software, which provides scientists with an easy and integrated system for the analysis of Affymetrix microarray experiments. AMDA is free and it is available as an R package. It is based on the Bioconductor project that provides a number of powerful bioinformatics and microarray analysis tools. This automated pipeline integrates different functions available in the R and Bioconductor projects with newly developed functions. AMDA covers all of the steps, performing a full data analysis, including image analysis, quality controls, normalization, selection of differentially expressed genes, clustering, correspondence analysis and functional evaluation. Finally a LaTEX document is dynamically generated depending on the performed analysis steps. The generated report contains comments and analysis results as well as the references to several files for a deeper investigation.

**Conclusion:**

AMDA is freely available as an R package under the GPL license. The package as well as an example analysis report can be downloaded in the Services/Bioinformatics section of the Genopolis

## Background

Microarrays have become common tools in many life-science laboratories. Despite their diffusion, it is still not easy to analyze the huge amount of data generated by this powerful technology. Microarray data analysis is in fact a multi-step procedure, and an overwhelming amount of different published methods exist for each step. While the research community has yet to agree on a golden standard, some methods have already been shown to be more appropriate in some situations [1]. On one hand, biologists that need to analyze their own microarray dataset may lack the necessary computational and statistical knowledge to address all aspects of a typical analysis work-flow. On the other hand, service providers that provide data analysis support to their user, have to face the challenge of transferring all the generated results in a comprehensive way and explaining the data analysis methods used at each step.

Many commercially or freely available tools exist to perform the common steps for analyzing microarray data. Some tools were developed for a particular or a very limited set of tasks [2,3], other tools try to cover many of the most important steps in data analysis [4-9]. The former rely on dealing with tool-specific input and output data formats, the latter do not provide an automated pipeline. In both cases multiple decisions are required and little documentation is provided to help interpret the results. Thus specific expertise are strongly recommended for a meaningful analysis. In addition, some client-server tools exist [10,11], that allow for visual composition of bioinformatics tools, plus Internet searches of publicly available components (by means of Web services technology). However the price to pay for such high flexibility and computational power is the complexity of software installation and maintenance, which practically require information technology specialists.

The only solution currently available to address most of the above issues is GenePublisher [12]. Unfortunately, it is only available as a web accessible service and it has a strong limitation on the number of samples that can be analyzed simultaneously. To date, input data must be included in a public database to get access to a server without input size restrictions.

None of the above mentioned methods allow the user to tailor the selection of differentially expressed genes (DEG) based on the experimental design and on the number of available replicates, while both criteria must be considered for a well-advised choice of the methods to use. Moreover, none of the above methods allow for the comparison of the results obtained with different DEG selection methods. In addition, none of them give the opportunity to incorporate *a priori *biological knowledge, e.g. in the form of user-defined gene lists, with the exception of Expression profiler [5]. Nevertheless the tool implemented in Expression profiler is based on the assumption that the genes belonging to the submitted families are co-expressed. Indeed genes that do not meet this prerequisite are removed while other co-expressed genes may be added in the original list(s). Finally, none of them, with the exception of GenePublisher [12], guide the user towards the interpretation of the results.

For all these reasons, we developed an R package that performs a fully automated microarray data analysis, in which the choice of the applied methods depends on the number of samples, on the experimental design and on the number of available replicates. It is based on standard or widely accepted methods [1] and relies on their R implementation. It allows a researcher with minimal computational skills to easily analyze his/her own data or data present in public repositories. This tool can be efficiently used to generate a first draft analysis, that could guide a more refined, specific analysis on relevant findings.

## Implementation

AMDA is a package entirely written in the R language [13,14]. The R/Bioconductor project [15] is becoming the reference open source software project for the analysis of genomic data. Moreover, R/Bioconductor is easy to install on the most common Operating Systems (Linux, Mac Os X, Windows).

Bioconductor developers created specific classes and methods for storing and dealing with microarray data (in particular the *exprSet *and *phenoData *objects). The AMDA package widely uses Bioconductor classes, therefore more experienced users can easily invoke other R/Bioconductor components in AMDA and vice-versa. In particular, the *exprSet *object is used for storing the expression intensities and it is strictly associated to the *phenoData *object used to document the experimental design.

Every function of the AMDA library is available as a stand-alone R tool and detailed help is available according to the standard format of R documentation. Moreover, the widget manual shows the few steps necessary for performing a complete analysis from image files via widget interface. Finally, a vignette (R interactive documentation format) is available to dynamically show a possible application of several functions contained in the AMDA package. It briefly shows how experienced R users can customize the pipeline, based on the order in which the different functions are applied and the setting of their parameters.

## Results and discussion

### The test dataset

AMDA was tested on a subset of a previously analyzed and published microarray dataset [16]. The report obtained with AMDA is available in an additional PDF file that can be downloaded from the Genopolis website (AMDAexampleReport.pdf) and some of its figures have been commented on the present paper. Briefly, the test dataset is the result of two time-course experiments. Mouse dendritic cells have been infected with two different forms of the *Leishmania mexicana *parasite. The first strain (pro) is the wild-type promastigote parasite, the second one (ko) is the same parasite knocked-down in the LPG1 gene necessary for the biosynthesis of one of the most important proteins expressed on the parasite surface (LPG). Four time points were obtained, nevertheless in this example only the first two were considered (4 and 8 hours). The first experiment includes untreated dendritic cells (NT, 3 replicates) and the samples harvested after infection for 4 and 8 hours with the pro parasite (pro4h, two replicates, and pro8h, two replicates, respectively). The second experiment includes the same baseline NT and samples harvested after infection for 4 and 8 hours with the ko parasite (ko4h, two replicates, and ko8h, two replicates, respectively).

The results obtained with AMDA are briefly discussed in the next sections compared to the findings previously published with in vitro and in vivo validation [16].

### The wrapper function and the widget interface

The wrapper function provides support to execute a specific work-flow. This function coordinates the set of available tools based on the required tasks (if specified) and available data.

**Figure 1:**
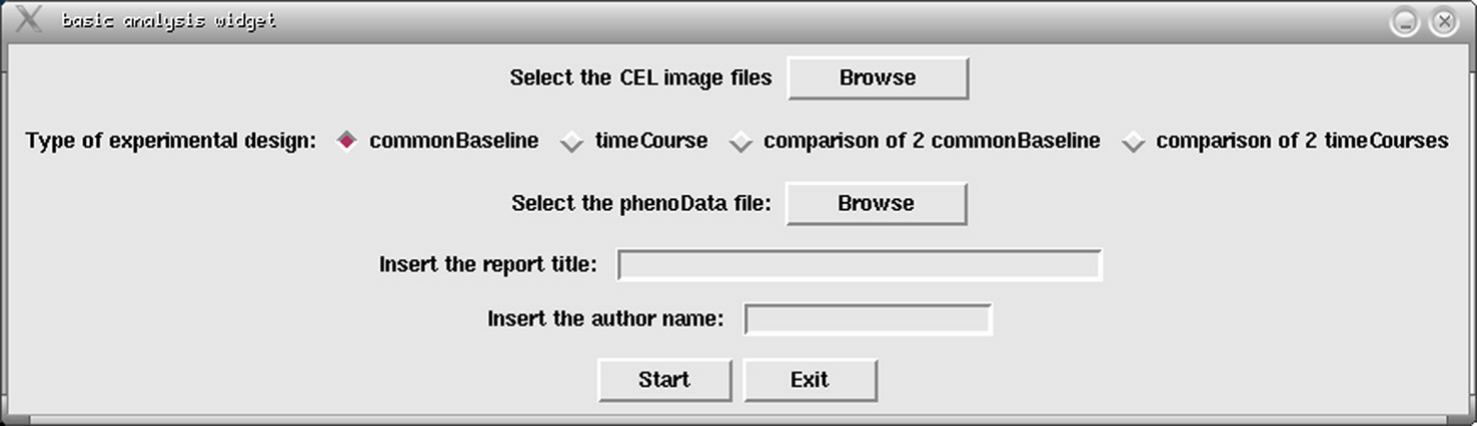
***The basic analysis widget***. This simple widget allows to run a complete analysis only prompting for few information. The list of CEL files, the type of experimental design and the phenodata file, that is necessary to assign each file to the respective condition, are required. Optionally the name of the report file and of the author can be provided.

**Figure 2:**
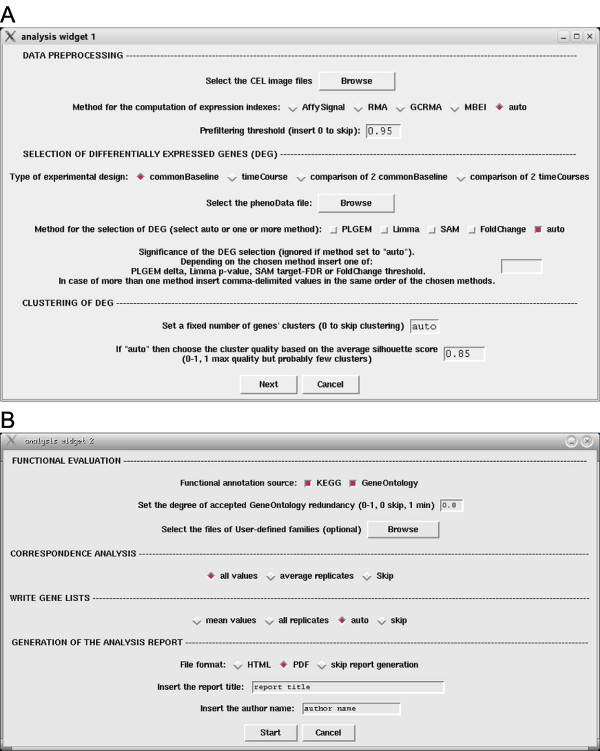
***The advanced analysis widgets***. Two widgets prompt the necessary information to run a complete analysis. These also allow the tuning of almost all the modifiable parameters of the tools available in the pipeline. Note that, when the *auto *option is available, there is the possibility to let AMDA decide the most appropriate setting. Basically the two widgets cover the loading and low level analysis of starting data, the selection of differentially expressed genes, their clustering (A), functional evaluation, correspondence analysis and setting of options on writing of gene lists and report files (B).

The easiest way to run the analysis is to use the *tcltk *widget interface. A basic and an advanced widget interfaces are available. If the former is chosen a very limited amount of inputs is prompted (Figure 1). Otherwise it is possible to use an advanced widget interface allowing the definition of almost all of the possible settings, in a user-friendly way (Figure 2).

The analysis can start from Affymetrix [17]*CEL *image files, that will be analyzed generating the expression indexes, i.e. the expression values for each probeset. Alternatively, the expression indexes can be stored and passed to AMDA functions through an *exprSet *object.

### Data preprocessing

Once the starting data are loaded some preprocessing functions are applied to perform quality checks, normalization and filtering. This part of the pipeline is also based on the *affy *and *gcrma *Bioconductor libraries.

The applied methods depend on the provided data. For example, quantile array normalization [18] is used if *CEL *image files are provided and the *RMA *algorithm [19] is required. However, if the number of samples is small, the Affymetrix algorithm is applied and the *Qspline *method [20] is used for the normalization of arrays. The reason for this choice is that the widely used quantile method is very useful if it is applied on probe-level data, but it makes the array distributions artificially similar if it is applied on summarized probeset data. In the latter case, a smoother normalization such as the *Qspline *method is more indicated [20].

Quality checks are performed for Affymetrix datasets to summarize global intensities of arrays and 5'/3' ratios of house-keeping control genes. The former data are useful to evaluate the average quality of the hybridization and the latter to assess the efficiency of *in vitro *cRNA synthesis reactions.

Filtering of data is optionally performed to eliminate data that are likely to be noisy. Probesets that are always called Absent are eliminated. Moreover a threshold is determined as the 95^th ^(default value that can be modified) percentile of the overall distribution of expression values called Absent. Probesets whose maximum value is below this threshold are also removed.

### Experimental design and selection of DEG

Four types of experimental design are considered:

1. *common baseline*: each treatment is compared to a common baseline;

2. *time-course*: each time point is compared to the previous one in the provided order;

3. comparison of two *common baseline *experiments: in each of the two experiments DEG are selected as in 1, additionally, the selection of DEG differently modulated between the two experiments is performed;

4. comparison of two *time-course *experiments: in each of the two experiments DEG are selected as in 2, additionally, the selection of DEG differently modulated between the two experiments is performed.

Four method are available for the selection of DEG, according to the chosen experimental method and number of available replicates: Limma, SAM, PLGEM and empirical Fold Change.

Limma ([21], based on the Bioconductor *limma *library) and SAM ([22], based on the Bioconductor *siggenes *library) can be applied only if replicates are provided for every condition. Limma can be used for all experimental designs while SAM can be applied only in the first two. PLGEM ([23], based on the Bioconductor *plgem *library) can be applied only with experimental design 1 and it is useful when none or few replicates are available for every condition except one. The empirical Fold Change method can be applied in case no, or few, replicates are available for every condition.

The wrapper function can autonomously choose the most appropriate method to use, based on the experimental design and the replication scheme. In this case the significance level of the selection is set by default depending on the chosen method. This is not a common feature in software where alternative methods are provided. Nevertheless, it is possible to force this setting and choose one of the available methods. In this case the significance level has to be set too, according to the selected method. For the *SAM *method it represents the target false discovery rate (FDR). In reality, *SAM *does not allow direct control of the FDR but it provides a table containing an estimated FDR for each tested parameter *delta*. AMDA uses this table for determining the *delta *value, giving the estimated FDR that is closest to the target, a feature not implemented in the *siggenes *package.

Finally, it is possible to select more than one method. In this case only DEG identified by each one of the chosen methods would be considered.

We would like to point out that the automatic decision of the most appropriated method for the DEG selection, and the possibility to use a combination of more than one method are novel features for this kind of software.

In the test analysis a comparison between two time courses was required. The Limma method was used for the selection of DEG in each time course and genes differently differentially expressed between the two time courses. The overall DEG universe is represented in a heat-map and the genes differently modulated in the two time courses are highlighted in gold (Figure 10 in the Additional file 1).

### Clustering of arrays and genes

Hierarchical clustering of arrays is performed to evaluate quality of replicates and qualitatively asses the degree of differential expression. For this purpose standard agglomerative complete-linkage hierarchical clustering algorithm is used (R package *stats*) similarly to the work of Eisen *et al*. [24]. Pearson's correlation is chosen as the similarity measure.

In addition, the overall set of genes that are differentially expressed in at least one condition is clustered with a partition algorithm to a number of clusters fixed by the user or automatically decided. In the latter case, the optimal number of clusters is estimated using the average silhouette scores method [25]. AMDA searches for a compromise between quality of clusters (high average score) and ease of biological interpretation (high, but biologically meaningful, number of clusters), accordingly to an adjustable parameter as described in more details in the documentation of the function *silPam*. Finally the normalized expression profiles of selected genes are clustered, according to the selected number of clusters, using the permutation around medoids method (PAM, Figure 11 in the Additional file 1, [26] and R library *cluster*). The supplemental figure shows the clusters obtained with the test dataset, a subset of a previously analyzed and published dataset [16]. The results obtained agree with the findings of this publication, since two main classes of genes are expected without any more complicated gene expression pattern, i.e. genes up- and down-regulated during the time-course.

Using a partitioning algorithm allows the user to easily deal with cluster gene-lists, in contrast to hierarchical algorithms. This can be easier for the user and simplify the functional evaluation of clustering results. In addition, the use of silhouette scores, although not new, is not a common feature in this kind of software.

### Functional evaluation

All generated lists of DEG and all the obtained clusters of co-regulated genes are subsequently functionally evaluated, based on GeneOntology (GO, [27] and Bioconductor libraries *GO *and *GOstats*) and KEGG pathways ([28,29] and Bioconductor library *KEGG*) annotation terms. The hyper-geometric distribution is used to compute p-values for assessing their over-representation in the gene lists. The terms are ranked based on their p-value and top ranking terms are selected.

In addition, GO terms can be filtered to eliminate redundancy. This is achieved by eliminating a GO term if one of its children maps to a set of genes overlapping more than 80% (default value of an adjustable parameter) to its parent's one. This allows to maintain only the most specific functional terms and to limit the amount of information to be reported in the figures.

It is also possible, and strongly suggested, to incorporate user-defined gene lists in the analysis. This allows the user to evaluate groups of genes that are *a priori *expected to be involved. Sets of DEG for each condition are functionally classified by generating figures where the statistic of differential expression for the probesets annotated with each significant annotation terms are reported. The results indicate several highly significant GO terms that are fully compliant with the expected functional response for the *pro4h *sample of this dataset. In fact, up-regulation of immune response (GO:0006955) and cytokine activity (GO:0005125) families is well described in Aebischer *et al*. [16].

In addition, a graphical summary is produced where all the relevant annotation terms, identified in each set of DEG, are reported. A heat-map is generated based on the p-value of each term in each condition. With this representation it is possible to easily select terms specific for some condition and biological processes, as well as molecular pathways/complexes, that are similarly affected under many experimental conditions. Also in this case the results are in full concordance with those published in Aebischer *et al*. [16].

For example, many GO terms linked to the inflammatory response (GO:0006954) are mainly specific for pro4h samples (Figure 23 in the Additional file 1) indicating the lack of pro-inflammatory response in the cells infected with the LPG-knock-out parasite.

We would like to emphasize that the use of custom gene families and this method of summarizing the results of a functional enrichment analysis are novel features for a microarray data analysis pipeline.

### Correspondence analysis

If more than two experimental conditions are provided, the overall set of genes that are differentially expressed in at least one condition is used to perform a correspondence analysis ([30] and R library *MASS*). Briefly, this technique allows to reduce the multi-dimensionality of a microarray dataset from both the point of view of the samples and the genes. It results in a bi-dimensional plot where sample labels and gene labels are separated according to their similarity. Thus, it is possible to select genes that are likely to be very significant for a particular set of samples, not necessarily foreseen during the experimental planning phase. A function is provided for selecting set of genes based on their coordinates in this plot. The selected set of genes is for instance suitable for a functional evaluation.

### The report and other resulting data

Most of the tools implemented in the AMDA pipeline produce some outputs by means of images, tables and/or annotated gene lists. A dynamically generated LaTEX report embeds these outputs together with proper text explanations. We have chosen LaTEX, because it is easy to handle, it is quite common in scientific literature production, and a number of converters to other formats exist (PDF, PostScript, HTML). The text embedded in the report is based on a series of templates and aims to guide the user through the interpretation of the results.

Many figures reference to files where annotated list of genes allow for a deeper investigation. These lists were generated by means of tools available in the Bioconductor library *annaffy*. For example, among the DEG differentially responsive in the first time-point of the two test experiments, the 11 probesets annotated with the inflammatory response GO term (GO:0006954, Figure 21 in the Additional file 1) can be retrieved in the file ./DEG/GO/4hko-4hpro_GO0006954.txt. This tab delimited file contains annotations as well as raw data and detection calls for the selected genes.

Finally, the LaTEX document can be automatically converted in a PDF file that can be easily zipped with the gene lists and sent by e-mail (usually less than 1MB).

Moreover, the main intermediate results obtained at each step are automatically saved as R objects and can be loaded into the R environment for further computation with the additional functions available in the AMDA package or in other R/Bioconductor packages.

### Comparison of functionalities among AMDA and other software that perform a full microarray data-analysis

Table 1 provides the comparison of the tools available in AMDA with the functionalities provided by other software, both web-services and client applications. Only GenePublisher [12], Expression Profiler [5] and GEPAS [4] offer a full set of tools that covers a complete analysis of a microarray dataset. Nevertheless, automation is provided only by GenePublisher, but this web-service does neither allow to choose among different experimental designs nor to choose among methods for the selection of DEG. Therefore the pipeline implemented in GenePublisher is less flexible than AMDA even though it offers other useful tools.

Expression Profiler and GEPAS include numerous tools but require adjustment of many parameters. Finally, while Expression Profiler allows the transfer of generated data from a tool to another, GEPAS is more demanding. Indeed it provides many independent tools, not conceived to be in a work-flow.

## Conclusion

The AMDA package is one of the few free tools allowing a complete and fully automated analysis of an Affymetrix microarray dataset. It is suitable to all researchers who want to easily and quickly obtain a preliminary analysis from their own or publicly available datasets, no specific training is required. Therefore AMDA can be useful for either service provider or scientists without specific training in microarray data-analysis. Moreover, since AMDA is fully integrated in the Bioconductor environment, it can be used in conjunction with other Bioconductor packages as part of a wider analysis. For the same reason, the wide and growing number of available tools are easy to integrate in AMDA.

## Availability and requirements

AMDA is freely available as an R GPL package in the Services/Bioinformatics section of Genopolis 

Software and hardware requirements are almost all the same of R/Bioconductor, with the exception of system tools necessary for the conversion of the LaTEX report into PDF in case of the installation in Windows systems (these can be downloaded and installed as indicated in the widget manual). The overall analysis does not require particular hardware. The time needed to terminate a wrapper function invocation depends on the choice of tools and on the dimension of the dataset. For example the test dataset referred to in the user manual consisting of 14 arrays and 6 experimental conditions can be analyzed starting from the image files and using all the provided tools in 12 minutes with a PC equipped with P4 processor and 2GB RAM. The results of this example analysis are available on the web site and are in full concordance with the findings reported in the original publication [16].

## Authors' contributions

MP conceived the software, wrote the code and the manuscript. NP contributed in conceiving the software, writing the code and revising the manuscript. MF participated in conceiving the software and revising the manuscript. PRC coordinated the study. All authors read and approved the final manuscript.

## Supplementary Material

Additional File 1 AMDA example report. PDF file containing the analysis report obtained by running AMDA on the test dataset described in "The test dataset" section; some of its figures have been commented in the present paper. This file can be downloaded from the Services/Bioinformatics section of the Genopolis . Click here for file

## Figures and Tables

**Table 1:** Comparison of functionalities among AMDA and other software that perform a full microarray data-analysis The functionalities provided by AMDA are reported in the first column, with comment or list of methods or DBs where necessary. The remaining columns show whether other software that perform a full microarray data-analysis provided the same features. A plus indicate that the software provides it and, when applicable, the same set of methods. A minus indicates that the functionality is not provided. Text is used to report in case the provided features are different. Finally, italic text is used to identify functionalities for which the provided methods are a sub-set of those available in AMDA.

**AMDA**	**Gene Publisher**^12^	**Expression Profiler**^5^	**GEPAS**^4^	**Array Pipe**^6^	**Race**^7^	**Midaw**^8^	**Sykacek *et al*.**^9^
widget interface	+	a web page for each tool	a web page for each tool	a web page for each tool	a web page for each tool	a web page for each tool	-

analysis of CEL files (signal,rma,gcrma,mbei)	*mbei*	+	+	no Affymetrix	+	no Affymetrix	no Affymetrix

quality controls	-	-	+	+	+	+	-

non-linear array normalization (quantile, qspline)	*qspline*	*quantile*	+	+	*quantile*	-	house keeping and spike RNA

array hierarchical clustering	+	+	+	-	-	+	-

replicates' scatter plot	all pairs	-	*manually*	-	-	-	-

support of many experimental designs (common ref, time-course, 2 common ref, 2 time-course)	*only common reference*	*manually with limma*	two class or multi-class	-	*only two class comparison*	*only two class comparison*	*only balanced reference design*

method for DEG selection (plgem, sam, FC, limma)	t-test with BH correction	*limma*, t-test	t-test, anova, clear test	t-test and method specific for spotted array	empirical bayes	*FC*, t-test. *sam*	mixed anova model

selection of many DEG methods	-	-	-	-	-	-	-

DEG heat-map	+	+	*not linked to DEG selection*	-	-	*not linked to DEG selection*	-

gene normalization	+	+	+	-	-	+	-

DEG clustering	+	+	*not linked to DEG selection*	-	-	*not linked to DEG selection*	-

silhouette-evaluation of clusters	+	-	-	-	-	-	-

functional evaluation of clusters (GO, KEGG, user-families)	-	*GO *and signature algorithm	*not linked to clusters selection, no user-families*	-	-	-	-

functional evaluation of DEG (GO, KEGG, user-families)	GO, KEGG	*GO *and signature algorithm	*not linked to DEG selection, no user-families*	*only export of annotated gene lists*	*GO*	-	-

functional summary	-	-	-	-	-	-	-

writing of annotated gene lists	-	-	-	-	-	-	-

correspondence analysis (bi-plot of arrays and genes)	*PCA(only arrays)*	+	-	-	-	*PCA(only arrays)*	-

dynamic PDF generation	+	-	-	-	-	-	-

flexible work-flow	-	+	+	+	-	-	-

automation	+	-	-	-	-	-	-

other tools not available in AMDA, notes	array classification, promoter analysis, prediction of orphan function	support to not affy, chr.local and sequence analysis	numerous independent tools	-	-	discriminant analysis (PAM)	imputation of missing values
