# Supplementary material for: AMDA: an R package for the automated microarray data analysis
Source: BMC Bioinformatics. 2006 Jul 6;7:335. doi: 10.1186/1471-2105-7-335 (PMC1534071; doi:10.1186/1471-2105-7-335)
Supplement: Additional File 1 — AMDA example report. PDF file containing the analysis report obtained by running AMDA on the test dataset described in "The test dataset" section; some of its figures have been commented in the present paper. This file can be downloaded from the Services/Bioinformatics section of the Genopolis . [file 1471-2105-7-335-S1.pdf]

example analysis AMDA version 2.0.0

Mattia Pelizzola

March 13, 2006

Abstract

This automatic report aims to provide a preliminary data analysis, which it is not intended to be exhaustive. This basic analysis provides the basic information that is necessary to interpret a microarray experiment. The data of the different arrays are made comparable by means of normalization techniques and clustered to group similar arrays. Differentially expressed genes (DEG) are selected according to a significance level and clustered to group genes with similar expression profiles across the provided samples. DEG and clusters are then functionally characterized with several databases and annotation terms are ranked based on their statistical relevance. Finally a correspondence analysis is preformed aiming at highlighting the most relevant genes for each experimental condition. All the code is written in the R language, making use of dedicated packages (see www.r-project.org/, www.bioconductor.org/, Genome Biol 2004,5:R80). This pipeline was developed by the Genopolis consortium and the R library and user manual are available on the consortium website (www.genopolis.it)

Contents

1 Data preprocessing **3**
1.1 Quality checks 3
1.2 Array normalization 4
1.3 Prefiltering 9

2 Clustering of arrays **10**

3 Selection of differentially expressed genes **11**

4 Clustering of differentially expressed genes **16**

5 Functional Annotation **26**
5.1 KEGG annotation terms 27
5.2 GeneOntology annotation terms 33
5.3 User-provided annotation terms 40

6 Correspondence analysis **47**

List of Figures

1 Quality checks of sample preparation and hybridization 5
2 Box plot of probe-level array data before the normalization procedure 6
3 Box plot of arrays data after the normalization procedure . . . 7
4 Scatter plot of non redundant replicated arrays after the normalization procedure 8
5 Distribution of Absent (red) and Present (blue) expression values 9
6 Hierarchical clustering of arrays 10
7 Experimental design 11
8 DEG found in the different experimental conditions 13
9 DEG found in the different experimental conditions for the second experiment. 13
10 Heat map of DEG universe. Each probeset is differentially expressed in at least one condition. The log2 of the ratio between each value and the median of the row is reported on a red-blue (high-low) color scale.Probesets differently differentially expressed between the two experiments are highlighted in gold colour. 15
11 Clustering of DEG 18
12 KEGG annotation terms for the experimental condition 4hpro-0hpro. 28
13 KEGG annotation terms for the experimental condition 8hpro-4hpro. 29
14 KEGG annotation terms for the experimental condition 4hko-0hko. 30
15 KEGG annotation terms for the experimental condition 4hko-4hpro. 30
16 KEGG annotation terms for the experimental condition 8hko-8hpro. 31
17 KEGG functional summary 32

18 GO annotation terms for the experimental condition 4hpro-0hpro. . . . 34
19 GO annotation terms for the experimental condition 8hpro-4hpro. . . . 35
20 GO annotation terms for the experimental condition 4hko-0hko. 36
21 GO annotation terms for the experimental condition 4hko-4hpro. 37
22 GO annotation terms for the experimental condition 8hko-8hpro. 38
23 GO functional summary 39
24 USER annotation terms for the experimental condition 4hpro-0hpro. . . . 41
25 USER annotation terms for the experimental condition 8hpro-4hpro. . . . 42
26 USER annotation terms for the experimental condition 4hko-0hko. . . . 43
27 USER annotation terms for the experimental condition 4hko-4hpro. . . . 44
28 USER annotation terms for the experimental condition 8hko-8hpro. . . . 45
29 USER functional summary 46
30 Correspondence analysis 48

List of Tables

1 Sample and condition names 4
2 KEGG annotation of cluster 1 19
3 KEGG annotation of cluster 2 19
4 KEGG annotation of cluster 3 20
5 GO annotation of cluster 1 20
6 GO annotation of cluster 2 21
7 GO annotation of cluster 3 22
8 USER annotation of cluster 1 23
9 USER annotation of cluster 2 24
10 USER annotation of cluster 3 25

1 Data preprocessing

1.1 Quality checks

Four quality checks are performed to verify the quality of sample preparation and hybridization (figure 1).

	timePoint	stimulus
NtA	0h	pro
NtB	0h	pro
NtC	0h	pro
Pro4hA	4h	pro
Pro4hB	4h	pro
Pro8hA	8h	pro
Pro8hB	8h	pro
kNtA	0h	ko
kNtB	0h	ko
kNtC	0h	ko
Ko4hA	4h	ko
Ko4hB	4h	ko
Ko8hA	8h	ko
Ko8hB	8h	ko

Table 1: Sample and condition names

First af all for each sample the percentage of probesets with Detection call "Absent" (A) over the total number of probesets is determined, as well as for the probesets with Detection call "Present" (P).

Next for each sample the mean value of probeset called A and P respectively is determined. Obviously the values associated to P calls are expected to be greater than values associated to A calls.

Finally the ratios between the expression values for 3' and 5' end of gapdh and actin transcripts are determined. This is useful to control the degree of sample degradation, since for these probesets probes are available to bind both ends of the transcript. If the in vitro synthesis reactions performed well the signal on both ends of the transcript is expected to be similar. Therefore a dashed horizontal line at the value of 1 represents the optimal performance.

1.2 Array normalization

In order to make the different chips (Table 1) comparable, it is necessary to normalize signal intensities. Two different diagnostic plots are useful for this purpose: box plots and scatter plots. Boxplots are one of the most intuitive ways of visualizing and comparing relevant properties of distributions of values across different arrays, e.g. first quartile, median and third quartile. Note that after normalization the distributions are more similar.

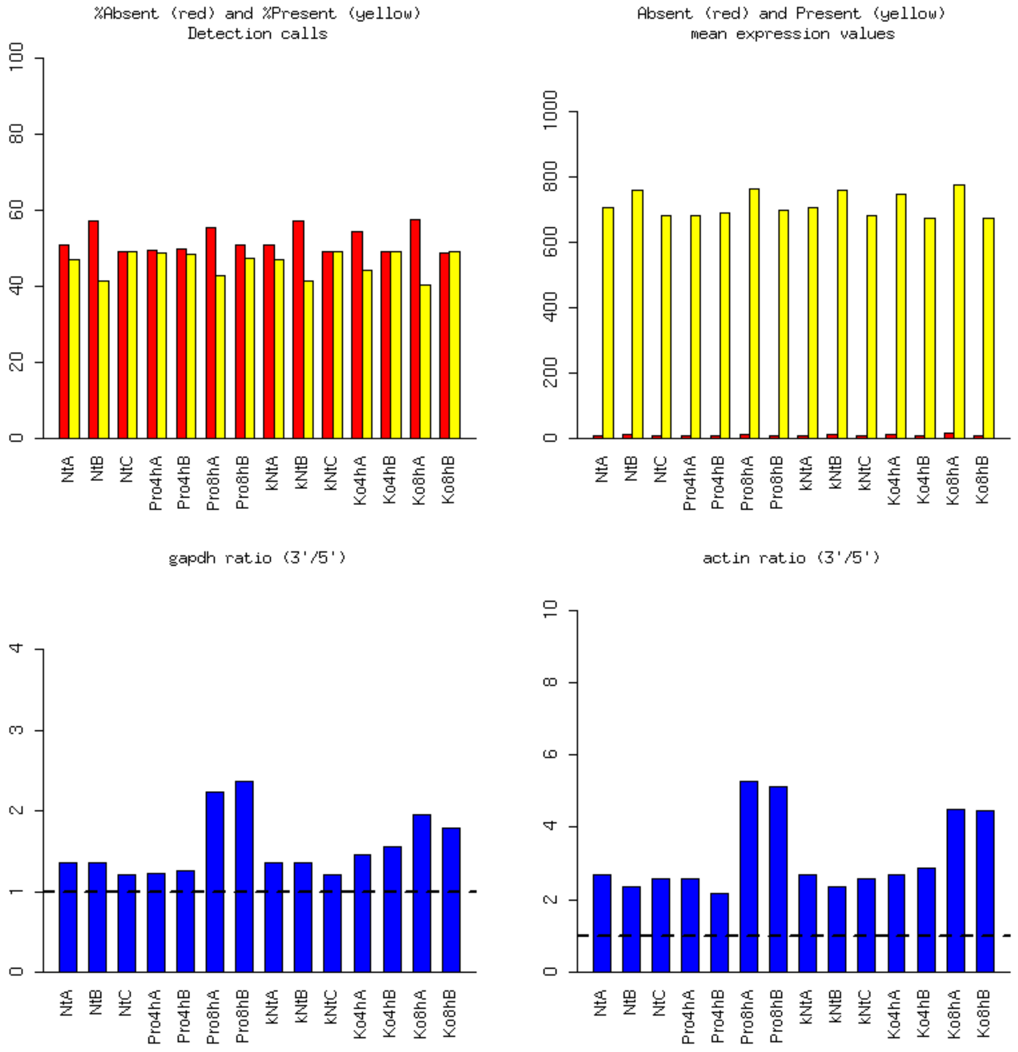

Figure 1: Quality checks of sample preparation and hybridization

Scatter plots are simply log plots of the expression intensities between for all the non redundant pairs of replicated samples. The adjusted R squared (adj-R2) measures goodness of fit of the respective linear regression in the scatter plots. In case of technical replicates an adj-R2 of around 0.95 is expected; in case of biological replicates the correlation can eventually drop below 0.9. Depending on the quality of the raw data at least a slight improvement of the correlation coefficient is usually observed after normalization.

Since the analysis started with the analysis of CEL image files, it is worth to normalize the samples at that level, i.e. at the probe level, before that

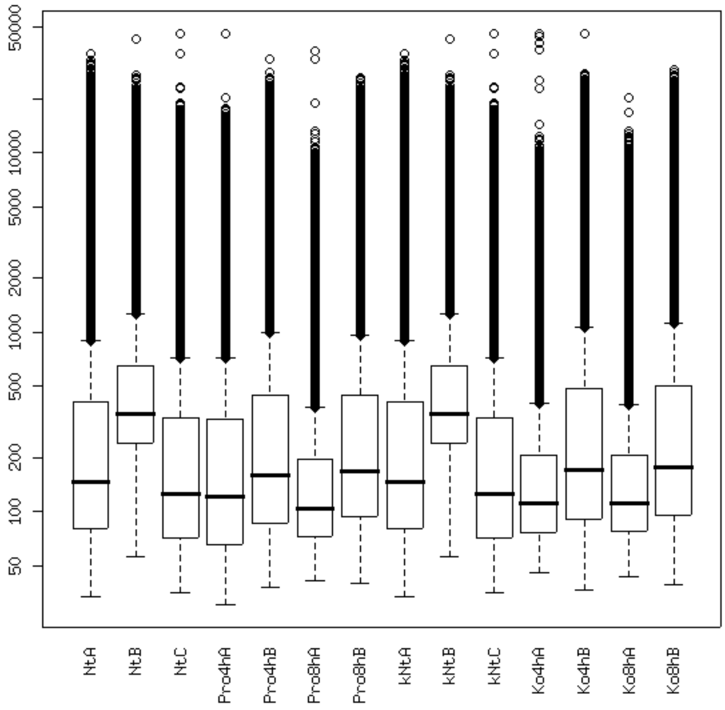

Figure 2: Box plot of probe-level array data before the normalization procedure

the different probes for each gene are summarized in one unique probeset. The figure 2 shows the boxplots for the distribution of probe level data in all the samples before the normalization procedure. Affymetrix Signal is used in case it is specifically required or in case the number of arrays is less than 7. If this algorithm is used the qSpline method is chosen for the normalization of samples after the generation of the probeset intensity summary (Workman et al., 2002). Other methods can be used if specifically required or if the number of arrays is greater than 7. These methods use the quantile method for the normalization of samples (Bolstad, 2001).

For this report probeset intensity summaries have been generated using GCRMA algorithm. The figure 3 shows the boxplots for the distribution of probeset intensities in all the samples after the normalization procedure. The figure 4 shows the logarithmic scatter plots of probeset intensities in all the non redundant pairs of replicated samples after the normalization procedure.

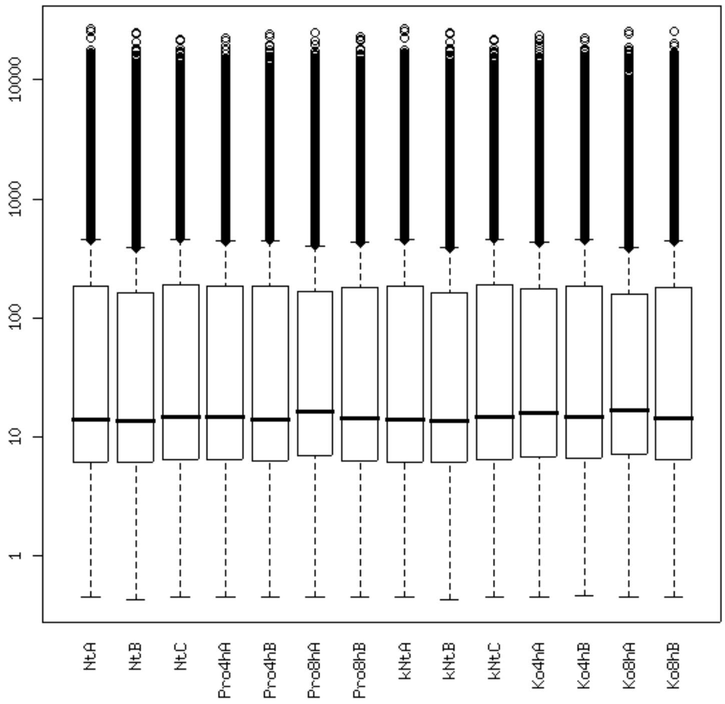

Figure 3: Box plot of arrays data after the normalization procedure

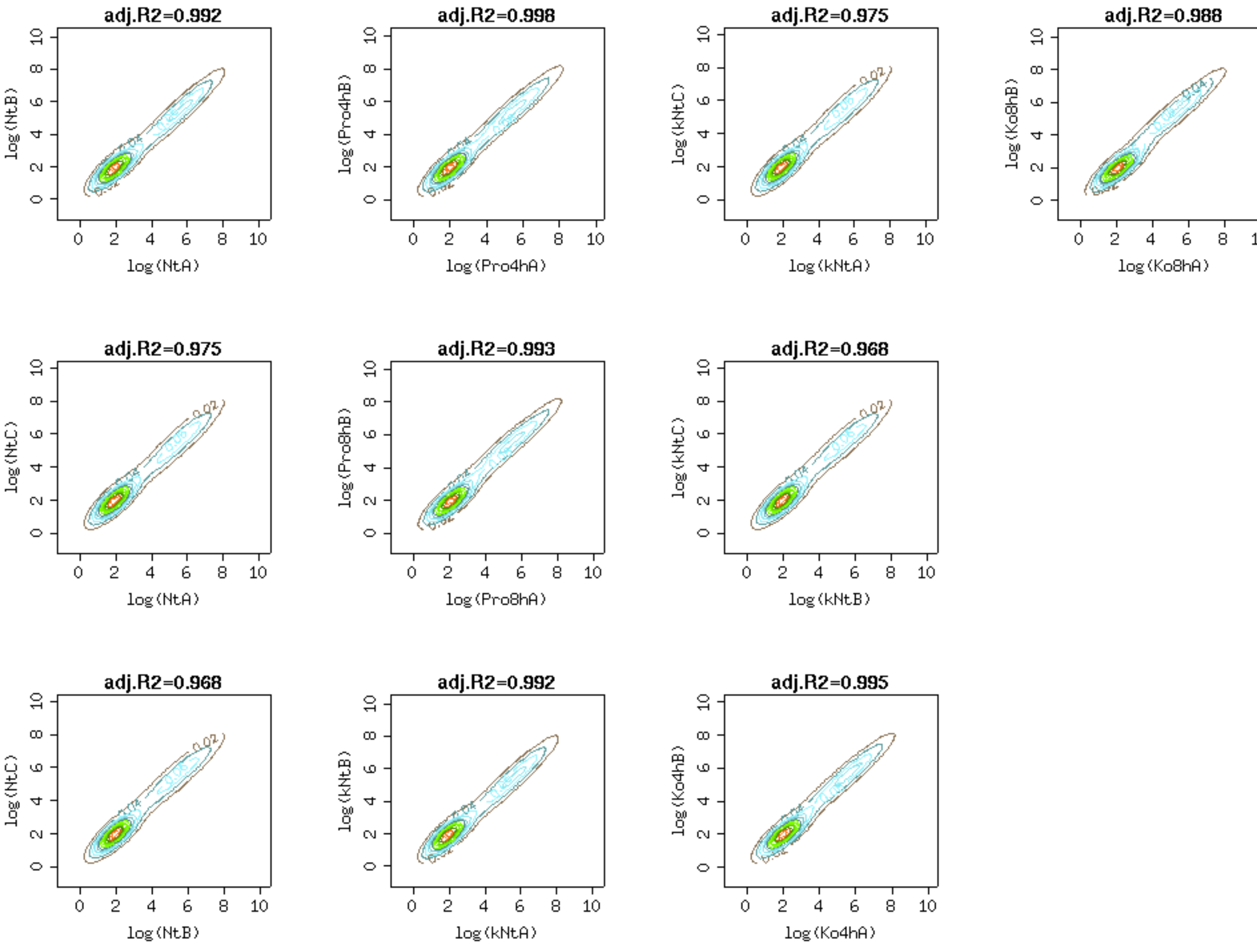

Figure 4: Scatter plot of non redundant replicated arrays after the normalization procedure

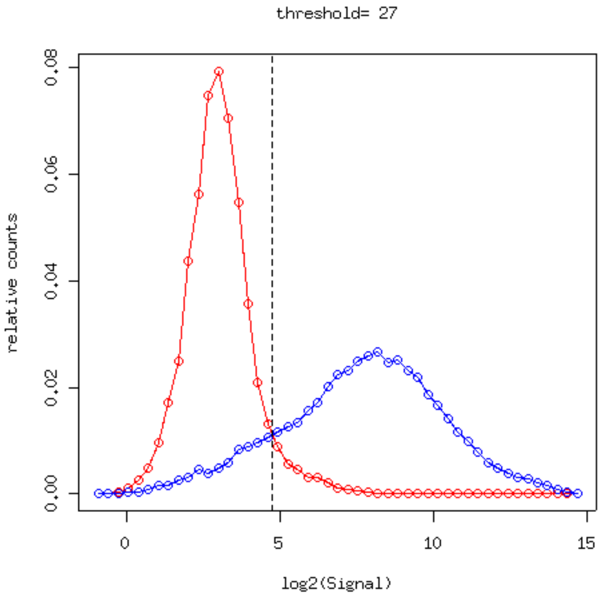

Figure 5: Distribution of Absent (red) and Present (blue) expression values

1.3 Prefiltering

To filter out noisy data before the selection of differentially expressed genes a filter is applied based on Detection calls. As a first step probesets called "Absent" (A) over all conditions and replicates are removed (5245). Indeed expression values flagged with an A call identify probesets whose PM values are not significantly different from MM control values. This indicates that the corresponding gene is either not expressed or that its expression can not be distinguished from noise. As a second step the 95th percentile of all the signals of the entire dataset that are flagged with an absent call is determined and used as a threshold to remove all the remaining probesets whose expression values are always below this value in each sample (1689). Finally 5554 probesets remain for the next analysis steps.

The figure 5 represents the distribution of all expression values flagged with an "Absent" call (A, red) and of all expression values flagged with a "Present" call (P, blue). The relative counts are the number of expression values within each interval normalized by the total number of P and A expression values. The log of expression values is reported and the dashed vertical line indicates the threshold used for the selection.

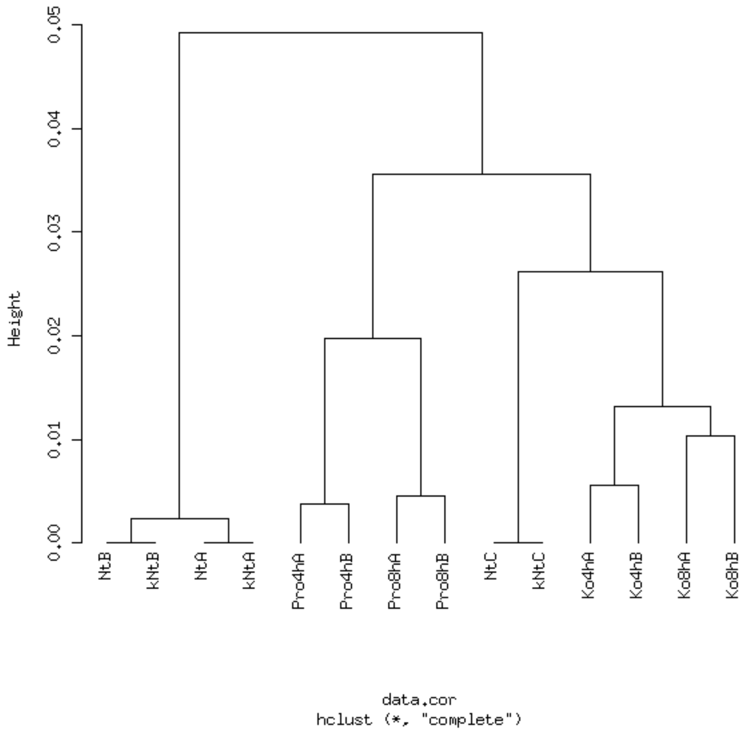

Figure 6: Hierarchical clustering of arrays

2 Clustering of arrays

We use a hierarchical clustering method to verify the quality of replicates and to highlight possible outlier samples which can be eventually excluded. By default, all chips are included for the further steps of the pipeline. The resulting dendrogram (figure 6) can be interpreted similarly to a phylogenetic tree. The vertical scale indicates 1 - pearson correlation coefficients as a measure of similarity. Replicates should cluster closer to each other than chips from different experimental conditions.

In addition, the resulting tree structure provides an unbiased overview on the relationship between the different experimental conditions. For example if three experimental conditions are investigated and all samples of exp.cond. 1 are closer to all samples of exp. cond. 2 than to exp. cond. 3, this means that the transcriptome of the cells is more similar between the first two conditions in comparison to the third one.

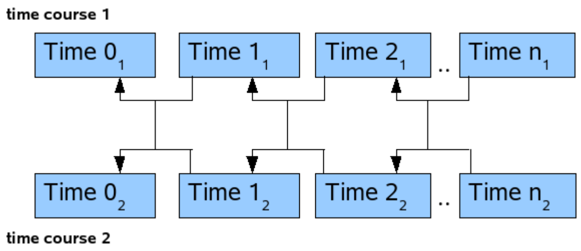

Figure 7: Experimental design

3 Selection of differentially expressed genes

The choice of the method used for the selection of differentially expressed genes (DEG) depends on the experimental design and on the number of available replicates for each conditions. Four experimental design are considered in AMDA:

1. common baseline (one or more treatment(s) have to be compared with the same baseline)

2. time course (each time point is compared to the previous one)

3. comparison of two common baseline (genes are selected as in 1 and if the are differently differentially expressed in respect to their respective baseline)

4. comparison of two time course (genes are selected as in 2 and if the are differently differentially expressed in respect to their respective previous time point).

For this report the comparison of 2 timeCourses experimental design has been selected (figure 7). Once the experimental design has been chosen, the choice of the method depends on the number of available replicates. Limma was used for the selection of DEG.

Limma is an acronym for Linear Models for Microarray Data (part of the following description is drew from the limma vignette). It is a Bioconductor library developed by Gordon Smyth et al (based on Gordon K. Smyth, Stat Appl Genet Mol Biol 2004). The use of this method is based on the fitting of a linear model to estimate the variability in the data. In case of one-channel microarray data (like Affymetrix) this approach is the same as analysis of variance except that a model is fitted for every gene.

For the detection of the differential expression an empirical Bayes method is used to moderate the standard errors. Indeed the use of moderated statistics for the detection of differential expression is very useful expecially in case of experiments with small number of replicates. Limma allows for the computation of moderated t-statistics for any required comparison across the conditions of the dataset, taking into account all the experimental designs previously reported in this report. In particular, among the methods available in AMDA, limma is the only method that can deals with experimental designs 3 and 4.

In this case all the requested comparisons were performed selecting DEG with a threshold pValue of 0.05.

The figure 8 represents with log scatter plots the DEG identified with the selected method(s). In these graphs the y-axis reports the mean of the replicates (if available) of the considered condition and the x-axis is the mean across the replicates of the reference condition. This reference condition is the baseline in case the commonBaseline experimental design was selected, the previous time-point condition in case the timeCourse experimental design was chosen. Red points are the DEG. Totally 1101 unique probesets have been selected (part of them can be differentially expressed in more than one condition, i.e. this number could not reflect the sum of DEG of the individual panels).

If you selected the option of writing files you can find in the folder DEG the deg.universe html and txt files containing a html and txt table with the probesets that were selected in at least one experimental condition. Moreover, in the same folder, you can find for each experimental condition a .txt tab delimited table reporting the identified DEG. This tables can be viewed using Excel and includes the most commonly used functional annotations, the normalized expression values with the corresponding detection Call, as well as the calculated statistic of differential expression for the considered experimental condition (STN for the PLGEM method, d-statistic for SAM, t-statistic for Limma, log2Ratio for FoldChange). An other similar figure is provided for the second experiment.

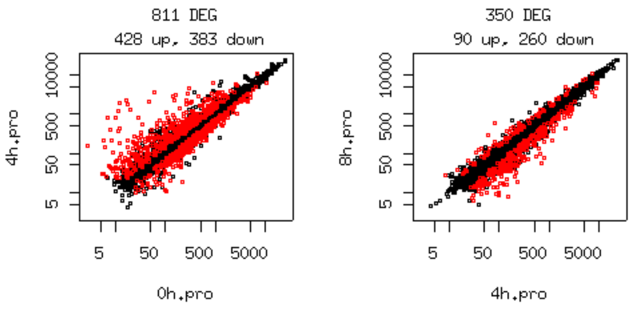

Figure 8: DEG found in the different experimental conditions

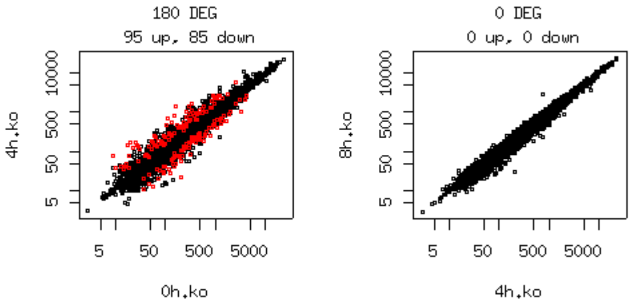

Figure 9: DEG found in the different experimental conditions for the second experiment.

The figure 10 is an heat map of the universe of DEG. For this figure the expression values of each probesets are divided for the median value and the log2 of this ratio is reported. Red colour indicates samples where the probeset is more expressed, blue colour samples where the expression is smaller. Probesets (rows) and samples (columns) are reordered based on their similarity, as indicated in the respective dendrograms.Since the experimental design 3 or 4 have been selected an additional colorbar for the rows is reported, where probesets differently differentially expressed between the two experiments are highlighted in gold colour.

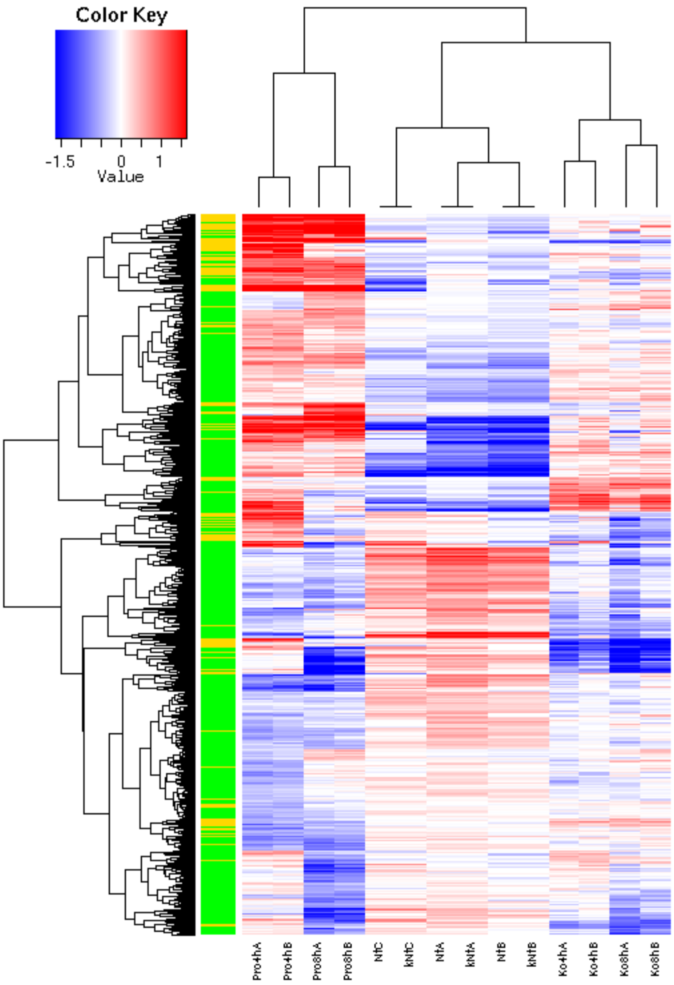

Figure 10: Heat map of DEG universe. Each probeset is differentially expressed in at least one condition. The log2 of the ratio between each value and the median of the row is reported on a red-blue (high-low) color scale.Probesets differently differentially expressed between the two experiments are highlighted in gold colour.

4 Clustering of differentially expressed genes

In experiments with at least three experimental conditions, clustering of the DEG is performed in order to group genes with similar expression profiles over all conditions. We use PAM-clustering method (Partitioning Around Medoid, Kaufman and Rousseeuw, 1990) to partition the gene expression profiles into k clusters. A fixed number of clusters can be chosen. Alternatively an estimate of the optimal k can be automatically determined, based on quality scores obtained from different trials of clustering.

Experimental conditions where replicates are available are averaged and the logarithm of the ratio between each exp. cond. and the condition chosen as the reference are calculated (log2Ratios). If the commonBaseline experimental design was selected, the reference is the baseline. Alternatively the log2 or the ratio with the median of the row for each probeset is computed.

The PAM clustering is repeatedly applied on the dataset testing various values of k between 2 and a number (6 or 10) that depends on the number of DEG. For each tested value of k a quality score (called "average mean silhouette") representing the averaged ratio between the within clusters similarity and the between-clusters dissimilarity is determined. The optimal number of clusters is generally determined as the one with the highest silhouette score. In case where there are more values of k that have very similar quality scores of silhouette close to the maximum one (0.85*max, this parameter can be set with or without the widget interface), the highest number of clusters is chosen. Anyway clusters with less than 10 probesets are not admitted.

The first panel (figure 11) represents the silhouette scores as a function of k. Based on this analysis 3 clusters were selected to partition the dataset. The remaining panels visualize the expression profiles of genes belonging to each cluster (log2Ratios). The red line represents the general pattern that characterizes each cluster (medoid). The similarity of the expression profiles of genes within a cluster suggests a similarity in their regulation. Even for poorly annotated genes (e.g. ESTs) a functional hint can be drawn from the occurrence of known genes within the same cluster.

If a functional analysis was requested , the clusters are evaluated for every available source of functional families (i.e. GeneOntology, KEGG or user-provided ones). A list of tables are embedded in the report containing the relevant annotation terms for each cluster and for each annotation source. If the option of writing files was chosen, the list of genes contained in each cluster is saved on the directory "Clusters". Moreover, for each annotation term in the tables a file is written in the directory "Clusters" and in the subdirectory corresponding to the annotation source (e.g. GO, KEGG, USER). The name of the file is composed of the number of cluster and the id of the

annotation term. The txt tab delimited files can be read using a spreadsheet program and contain the list of probesets in that cluster matching the selected annotation term. Moreover a useful list of external annotations and DB references is added, and also the normalized values and detection calls are provided. In this tables for each annotation term the number of annotated-probesets contained in the considered cluster is reported (id on list). the next column reports the number of probesets matching the considered annotation term on the whole array (id on chip). These two numbers give an idea on the significance of the functional enrichment of the cluster list. The last column reports the pValue generated by a statistical test applied with the null hypothesis that this enrichment is random. The more this pValue is low the more the enrichment is statistically significative. Generally only pValue very low are considered interesting in this test, e.g. 1e-5 or lower.

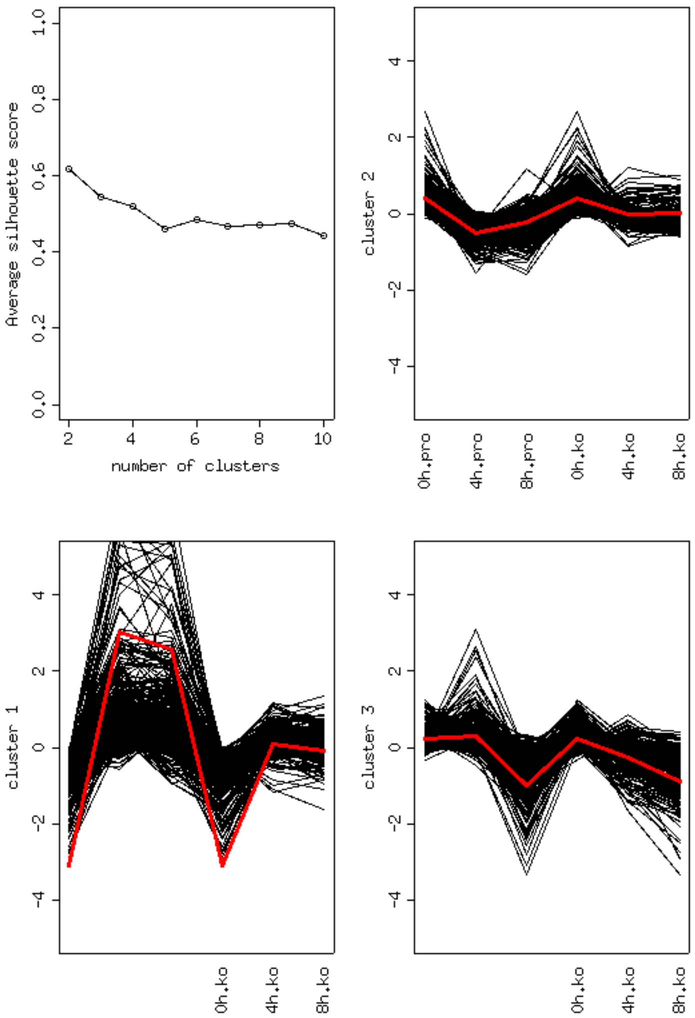

Figure 11: Clustering of DEG

	id	description	id on list	id on chip	pValue
1	04060	Cytokine-cytokine receptor interaction	38	245	3.99e-15
2	04620	Toll-like receptor signaling pathway	18	94	8.29e-10
3	04630	Jak-STAT signaling pathway	18	152	2.39e-06
4	00791	Atrazine degradation	3	6	2.4e-05
5	04640	Hematopoietic cell lineage	12	110	0.000167
6	04920	Adipocytokine signaling pathway	9	76	0.000392
7	04514	Cell adhesion molecules (CAMs)	13	160	0.00201
8	04210	Apoptosis	10	113	0.0026
9	00252	Alanine and aspartate metabolism	3	26	0.0135
10	04010	MAPK signaling pathway	16	268	0.0179
11	04660	T cell receptor signaling pathway	8	115	0.0236
12	02010	ABC transporters - General	3	31	0.0246
13	04530	Tight junction	8	120	0.03
14	04662	B cell receptor signaling pathway	5	73	0.0484
15	00760	Nicotinate and nicotinamide metabolism	3	39	0.0514
16	04512	ECM-receptor interaction	6	99	0.0675
17	00193	ATP synthesis	3	48	0.0949
18	00240	Pyrimidine metabolism	4	73	0.124
19	04610	Complement and coagulation cascades	4	79	0.158
20	04810	Regulation of actin cytoskeleton	8	235	0.48
21	00190	Oxidative phosphorylation	4	132	0.523
22	04520	Adherens junction	3	110	0.567
23	00230	Purine metabolism	3	117	0.616
24	04910	Insulin signaling pathway	3	138	0.741
25	04510	Focal adhesion	4	254	0.956
26	04080	Neuroactive ligand-receptor interaction	3	241	0.977

Table 2: KEGG annotation of cluster 1

	id	description	id on list	id on chip	pValue
1	04110	Cell cycle	14	107	7.43e-07
2	00604	Ganglioside biosynthesis	3	20	0.00272
3	00970	Aminoacyl-tRNA synthetases	3	22	0.00392
4	00640	Propanoate metabolism	3	23	0.00464
5	00230	Purine metabolism	8	117	0.00907
6	00600	Glycosphingolipid metabolism	3	33	0.0169
7	00240	Pyrimidine metabolism	5	73	0.023
8	00280	Valine, leucine and isoleucine degradati	3	41	0.0347
9	00561	Glycerolipid metabolism	3	44	0.0434
10	00620	Pyruvate metabolism	3	45	0.0465
11	00071	Fatty acid metabolism	4	68	0.0547
12	04910	Insulin signaling pathway	7	138	0.0584
13	04662	B cell receptor signaling pathway	4	73	0.07
14	00330	Arginine and proline metabolism	3	54	0.0802
15	00190	Oxidative phosphorylation	6	132	0.106
16	04070	Phosphatidylinositol signaling system	4	86	0.119
17	04920	Adipocytokine signaling pathway	3	76	0.198
18	04514	Cell adhesion molecules (CAMs)	6	160	0.212
19	04540	Gap junction	4	108	0.229
20	04510	Focal adhesion	9	254	0.241
21	04210	Apoptosis	4	113	0.257
22	04660	T cell receptor signaling pathway	4	115	0.269
23	04310	Wnt signaling pathway	5	150	0.302
24	04630	Jak-STAT signaling pathway	5	152	0.313
25	04620	Toll-like receptor signaling pathway	3	94	0.317
26	04020	Calcium signaling pathway	6	186	0.333
27	04060	Cytokine-cytokine receptor interaction	6	245	0.615
28	04010	MAPK signaling pathway	5	268	0.826
29	04810	Regulation of actin cytoskeleton	4	235	0.844

Table 3: KEGG annotation of cluster 2

	id	description	id on list	id on chip	pValue
1	00240	Pyrimidine metabolism	8	73	2.69e-05
2	04514	Cell adhesion molecules (CAMs)	11	160	0.000191
3	05050	Dentatorubropallidoluysian atrophy (DRPL	4	28	0.000303
4	04810	Regulation of actin cytoskeleton	13	235	0.000619
5	00740	Riboflavin metabolism	3	20	0.000799
6	05010	Alzheimer's disease	4	35	0.000883
7	04660	T cell receptor signaling pathway	8	115	0.000885
8	00230	Purine metabolism	8	117	0.001
9	03030	DNA polymerase	3	24	0.00164
10	05040	Huntington's disease	4	40	0.00164
11	01510	Neurodegenerative Disorders	4	51	0.00482
12	04662	B cell receptor signaling pathway	5	73	0.00499
13	04110	Cell cycle	6	107	0.00867
14	04520	Adherens junction	6	110	0.01
15	04010	MAPK signaling pathway	11	268	0.0142
16	04620	Toll-like receptor signaling pathway	5	94	0.0164
17	00193	ATP synthesis	3	48	0.02
18	04640	Hematopoietic cell lineage	5	110	0.0325
19	04210	Apoptosis	5	113	0.0364
20	04070	Phosphatidylinositol signaling system	4	86	0.039
21	00562	Inositol phosphate metabolism	3	63	0.0477
22	00564	Glycerophospholipid metabolism	3	67	0.0575
23	00190	Oxidative phosphorylation	5	132	0.0677
24	04510	Focal adhesion	8	254	0.102
25	04310	Wnt signaling pathway	5	150	0.108
26	04020	Calcium signaling pathway	6	186	0.111
27	04630	Jak-STAT signaling pathway	5	152	0.113
28	04530	Tight junction	4	120	0.12
29	04060	Cytokine-cytokine receptor interaction	7	245	0.164
30	04910	Insulin signaling pathway	3	138	0.352

Table 4: KEGG annotation of cluster 3

	id	description	id on list	id on chip	pValue
1	GO:0006952	defense response	72	418	2.03e-22
2	GO:0009607	response to biotic stimulus	77	492	2.49e-21
3	GO:0006955	immune response	60	338	2.66e-19
4	GO:0050896	response to stimulus	86	824	1.4e-12
5	GO:0009613	response to pest, pathogen or parasite	35	215	1.93e-10
6	GO:0043207	response to external biotic stimulus	36	227	2.23e-10
7	GO:0001664	G-protein-coupled receptor binding	12	30	4.43e-09
8	GO:0050874	organismal physiological process	69	718	1.56e-08
9	GO:0042379	chemokine receptor binding	11	28	2.5e-08
10	GO:0008009	chemokine activity	11	28	2.5e-08
11	GO:0006954	inflammatory response	19	92	6.85e-08
12	GO:0009605	response to external stimulus	48	462	3.71e-07
13	GO:0050776	regulation of immune response	14	62	1.21e-06
14	GO:0009611	response to wounding	24	167	1.68e-06
15	GO:0050778	positive regulation of immune response	12	48	2.25e-06
16	GO:0042089	cytokine biosynthesis	10	33	2.38e-06
17	GO:0042107	cytokine metabolism	10	33	2.38e-06
18	GO:0005125	cytokine activity	21	147	5.3e-06
19	GO:0051240	positive regulation of organismal physio	12	52	5.58e-06
20	GO:0001816	cytokine production	10	36	5.73e-06
21	GO:0042330	taxis	14	72	7.98e-06
22	GO:0006935	chemotaxis	14	72	7.98e-06
23	GO:0042035	regulation of cytokine biosynthesis	8	26	2.22e-05
24	GO:0009615	response to virus	8	26	2.22e-05
25	GO:0001817	regulation of cytokine production	8	27	3.02e-05
26	GO:0009891	positive regulation of biosynthesis	7	22	5.79e-05
27	GO:0006950	response to stress	42	467	7.66e-05
28	GO:0046649	lymphocyte activation	13	77	8.03e-05
29	GO:0051239	regulation of organismal physiological p	16	111	9.06e-05
30	GO:0051247	positive regulation of protein metabolis	8	32	0.000115
31	GO:0046651	lymphocyte proliferation	8	32	0.000115
32	GO:0009889	regulation of biosynthesis	12	70	0.00013
33	GO:0051251	positive regulation of lymphocyte activa	8	33	0.000146
34	GO:0050867	positive regulation of cell activation	8	33	0.000146
35	GO:0050671	positive regulation of lymphocyte prolif	6	18	0.00015
36	GO:0042221	response to chemical substance	14	95	0.000196
37	GO:0001819	positive regulation of cytokine producti	6	19	0.00021
38	GO:0042108	positive regulation of cytokine biosynth	6	19	0.00021
39	GO:0019955	cytokine binding	10	55	0.000225
40	GO:0001775	cell activation	13	85	0.000226

Table 5: GO annotation of cluster 1

	id	description	id on list	id on chip	pValue
1	GO:0005622	intracellular	187	4045	1.06e-07
2	GO:0006261	DNA-dependent DNA replication	8	26	2.46e-06
3	GO:0043227	membrane-bound organelle	150	3122	3.2e-06
4	GO:0043231	intracellular membrane-bound organelle	150	3122	3.2e-06
5	GO:0043226	organelle	165	3543	3.38e-06
6	GO:0043229	intracellular organelle	165	3543	3.38e-06
7	GO:0006259	DNA metabolism	27	296	8.34e-06
8	GO:0008094	DNA-dependent ATPase activity	6	18	3.07e-05
9	GO:0043283	biopolymer metabolism	83	1542	4.32e-05
10	GO:0007049	cell cycle	30	385	5.6e-05
11	GO:0015081	sodium ion transporter activity	7	32	0.000132
12	GO:0008137	NADH dehydrogenase (ubiquinone) activity	7	32	0.000132
13	GO:0003954	NADH dehydrogenase activity	7	32	0.000132
14	GO:0050136	NADH dehydrogenase (quinone) activity	7	32	0.000132
15	GO:0016655	oxidoreductase activity, acting on NADH	7	33	0.000162
16	GO:0046873	metal ion transporter activity	9	56	0.000185
17	GO:0006270	DNA replication initiation	4	9	0.000188
18	GO:0003824	catalytic activity	124	2600	0.000189
19	GO:0031324	negative regulation of cellular metaboli	13	114	0.000238
20	GO:0000074	regulation of progression through cell c	19	214	0.000275
21	GO:0019886	antigen processing, exogenous antigen vi	4	10	0.000305
22	GO:0044237	cellular metabolism	152	3484	0.000544
23	GO:0050875	cellular physiological process	201	4945	0.000554
24	GO:0044238	primary metabolism	146	3325	0.000637
25	GO:0005634	nucleus	96	1972	0.000723
26	GO:0009892	negative regulation of metabolism	14	144	0.000723
27	GO:0006260	DNA replication	10	82	0.000733
28	GO:0016651	oxidoreductase activity, acting on NADH	7	42	0.000772
29	GO:0006631	fatty acid metabolism	10	84	0.000887
30	GO:0048523	negative regulation of cellular process	24	334	0.00104
31	GO:0044249	cellular biosynthesis	36	585	0.00113
32	GO:0009058	biosynthesis	39	653	0.00122
33	GO:0006633	fatty acid biosynthesis	6	34	0.00126
34	GO:0030554	adenyl nucleotide binding	42	713	0.0014
35	GO:0046394	carboxylic acid biosynthesis	6	35	0.00147
36	GO:0016053	organic acid biosynthesis	6	35	0.00147
37	GO:0030333	antigen processing	5	24	0.00149
38	GO:0005524	ATP binding	41	698	0.0017
39	GO:0048519	negative regulation of biological proces	25	367	0.00175
40	GO:0005739	mitochondrion	32	510	0.00191

Table 6: GO annotation of cluster 2

	id	description	id on list	id on chip	pValue
1	GO:0046649	lymphocyte activation	10	77	0.000153
2	GO:0006259	DNA metabolism	22	296	0.000178
3	GO:0001837	epithelial to mesenchymal transition	3	5	0.000308
4	GO:0001775	cell activation	10	85	0.00035
5	GO:0045321	immune cell activation	10	85	0.00035
6	GO:0000166	nucleotide binding	49	1003	0.000354
7	GO:0042110	T cell activation	7	43	0.000376
8	GO:0017076	purine nucleotide binding	44	895	0.000668
9	GO:0050875	cellular physiological process	177	4945	0.000831
10	GO:0009262	deoxyribonucleotide metabolism	3	7	0.00103
11	GO:0006260	DNA replication	9	82	0.00115
12	GO:0007582	physiological process	191	5500	0.00129
13	GO:0045012	MHC class II receptor activity	3	8	0.00138
14	GO:0016462	pyrophosphatase activity	19	293	0.00138
15	GO:0016817	hydrolase activity, acting on acid anhyd	19	295	0.0015
16	GO:0016818	hydrolase activity, acting on acid anhyd	19	295	0.0015
17	GO:0051084	posttranslational protein folding	3	8	0.00161
18	GO:0051085	chaperone cofactor dependent protein fol	3	8	0.00161
19	GO:0009986	cell surface	8	75	0.00162
20	GO:0006952	defense response	25	418	0.0017
21	GO:0019882	antigen presentation	5	30	0.00237
22	GO:0007049	cell cycle	23	385	0.00265
23	GO:0019886	antigen processing, exogenous antigen vi	3	10	0.00328
24	GO:0005524	ATP binding	34	698	0.00347
25	GO:0009607	response to biotic stimulus	27	492	0.00381
26	GO:0042113	B cell activation	5	34	0.00418
27	GO:0017111	nucleoside-triphosphatase activity	17	277	0.00434
28	GO:0030554	adenyl nucleotide binding	34	713	0.00486
29	GO:0001776	immune cell homeostasis	3	12	0.00574
30	GO:0042591	antigen presentation, exogenous antigen	3	12	0.00574
31	GO:0030333	antigen processing	4	24	0.00655
32	GO:0001894	tissue homeostasis	3	13	0.00729
33	GO:0051301	cell division	9	109	0.00795
34	GO:0051249	regulation of lymphocyte activation	5	40	0.00847
35	GO:0050865	regulation of cell activation	5	40	0.00847
36	GO:0005623	cell	178	5596	0.00909
37	GO:0009897	external side of plasma membrane	6	61	0.00913
38	GO:0006955	immune response	19	338	0.0116
39	GO:0050896	response to stimulus	38	824	0.0118
40	GO:0005525	GTP binding	12	189	0.0122

Table 7: GO annotation of cluster 3

	id	description	id on list	id on chip	pValue
1	7	Chemokines	13	34	3.89e-12
2	16	Inflammatory response	17	76	1.47e-10
3	22	NF-kB	6	23	1.15e-05
4	6	Chemokine receptors	5	21	7.42e-05
5	9	Costimulation	4	16	0.000191
6	19	Interleukins	5	27	0.000337
7	18	Interleukin receptors	5	31	0.000741
8	20	JAK-STAT-SOCS	3	23	0.00868
9	26	Protein kinases	25	437	0.00898
10	2	Apoptosis induction	4	39	0.0127
11	13	Growth factors	9	142	0.0335
12	10	Cystein proteases	6	101	0.0734
13	4	Apoptosis (other)	8	158	0.12
14	28	Serine proteases	7	150	0.177
15	31	Ubiquitin pathway	4	91	0.233
16	21	Metallo peptidases	4	104	0.324
17	27	Protein phosphatases	3	114	0.596
18	12	G-protein coupled receptors	7	234	0.616
19	5	Cell adhesion	10	400	0.863

Table 8: USER annotation of cluster 1

	id	description	id on list	id on chip	pValue
1	1	Antigen processing and presentation	5	32	0.000344
2	26	Protein kinases	20	437	0.025
3	10	Cystein proteases	4	101	0.191
4	27	Protein phosphatases	4	114	0.263
5	31	Ubiquitin pathway	3	91	0.297
6	4	Apoptosis (other)	5	158	0.345
7	21	Metallo peptidases	3	104	0.386
8	5	Cell adhesion	4	400	0.994

Table 9: USER annotation of cluster 2

	id	description	id on list	id on chip	pValue
1	1	Antigen processing and presentation	5	32	5.57e-05
2	16	Inflammatory response	7	76	0.000233
3	14	Heat shock proteins	4	43	0.00228
4	26	Protein kinases	15	437	0.029
5	27	Protein phosphatases	4	114	0.102
6	25	Other proteases	3	87	0.12
7	12	G-protein coupled receptors	5	234	0.398
8	4	Apoptosis (other)	3	158	0.45
9	5	Cell adhesion	4	400	0.938

Table 10: USER annotation of cluster 3

5 Functional Annotation

A functional annotation of DEG is performed on the basis of a subset of the annotation provided by the Bioconductor project (mgu74av2 1.10.0 from www.bioconductor.org). The annotation resources considered depend on the organism and generally include GeneOntology and KEGG (www.geneontology.org, www.genome.jp/kegg).

The analysis of user defined functional families is always strongly suggested. These lists of probesets can be easily added to the analysis starting with the advanced widget interface. More files each one with a column of probesets ids of the right chip are expected. Their analysis should provide information about functional terms known to be (or not to be) involved in the biological process under investigation. The user defined functional families can be some lists of genes that can not be easily generated with the common annotation databases (e.g. GeneOntology and Kegg are under continuous update..), or can even be list of genes taken from a review or a table of an interesting paper, that you want to evaluate in your experiment.

On the other side KEGG and GO terms could provide new unexpected functional hints since they are evaluated without a priori selection.

The most representative functional annotations for DEG from each experimental condition are identified by determining the probability of random occurrence of functional terms (functional enrichment). Based on this probability ranking only the top 40 statistically most significant annotation terms are reported, and the most interesting are red highlighted. Each figure reports the selected annotation terms for the considered experimental condition. For each annotation term the following information is provided: annotation term ID, annotation term description, number of DEG in that condition annotated with the term, number of probesets on the whole chip annotated with the term, pValue of functional enrichment. The modulation of genes for the selected annotation terms is visualized for each experimental condition and the x-axis of each figure. To perform a statistical test not biased by the redundancy of the probesets (more probesets for the same gene), the computation of pValue of functional enrichment is based on entrezGene IDs. Since more probesets could match to a single entrezGene ID, the number of DEG annotated with the term can be less than the number of probesets visualized (the blue circles).

If at least two experimental conditions are provided a functional summary is provided reporting the enrichment pvalues (log10 of pValues) for the different annotation terms across the different conditions. This plot can be useful to compare the functional characterization of DEG in the different conditions. In particular annotation terms specific for a subset of conditions

could be identified, as well as annotation terms that are equally relevant for all the conditions. Note that the colours of this graph reflect only the enrichment pvalues (highly significative is red), they are not representative of the direction of the modulation (up, down-modulated). Therefore genes with annotation terms equally significant in more conditions could be differently modulated (this can be evaluated with the figures specific for each condition).

If the write files option was chosen, for each annotation term in each figure, the considered DEG are reported on a tab delimited .txt file structured as described in the section reporting the selection of DEG.

5.1 KEGG annotation terms

Kyoto Encyclopedia of Genes and Genomes (KEGG) provides the reference collection of metabolic pathways. In the currently used database built of the Bioconductor annotation package about hundred pathways are considered (KEGG library, version 1.10.0). Only pathways represented with at least 2 DEG are ranked according to their relevance. Based on this ranking only the first 40 (or less whenever this number is not reached) are reported, for plotting reason. Figures from 12 to 16. If at least two experimental conditions are provided a functional summary is provided (17) reporting the enrichment pvalues (log10 of pValues) for the different annotation terms and their similarity.

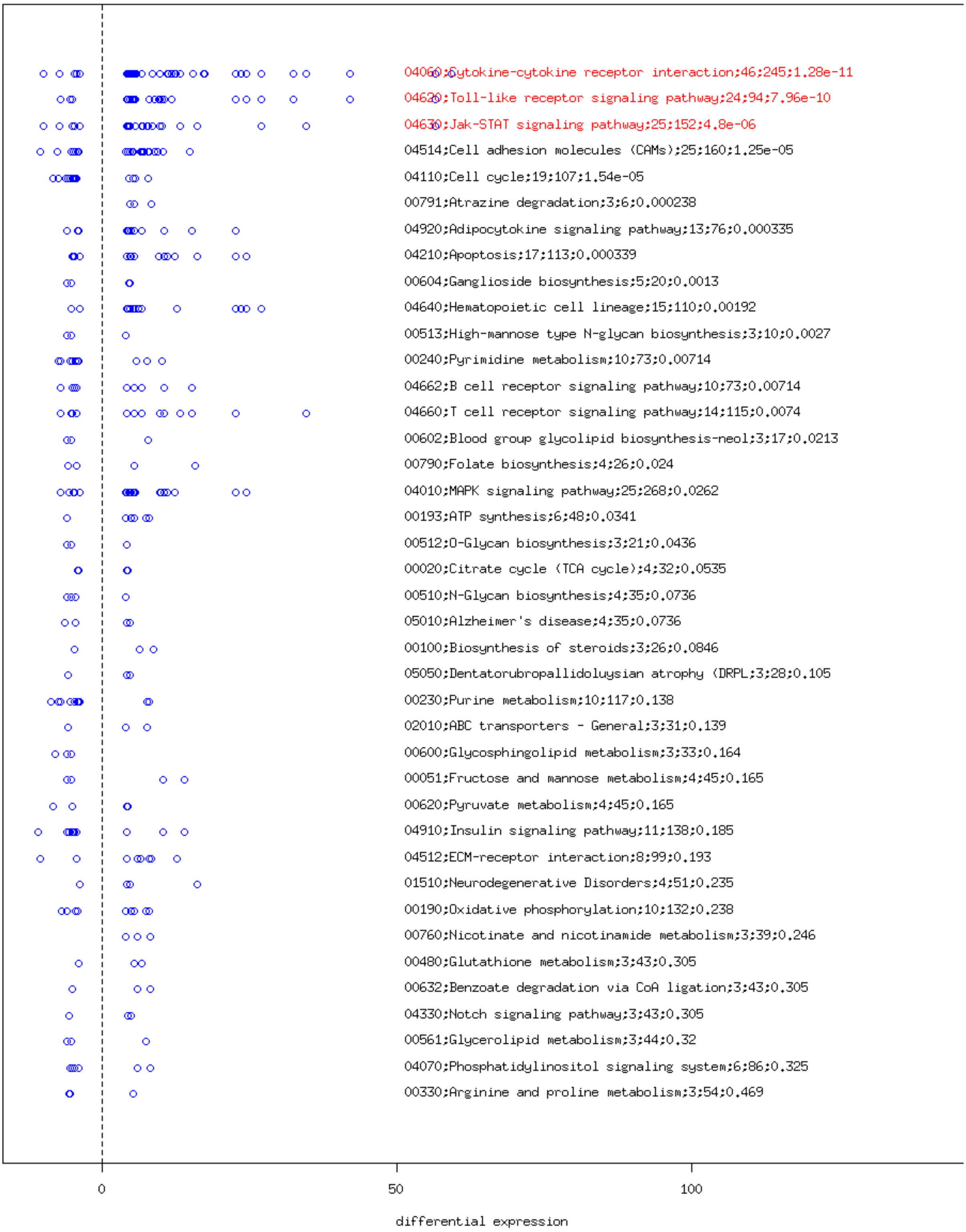

Figure 12: KEGG annotation terms for the experimental condition 4hpro-0hpro.

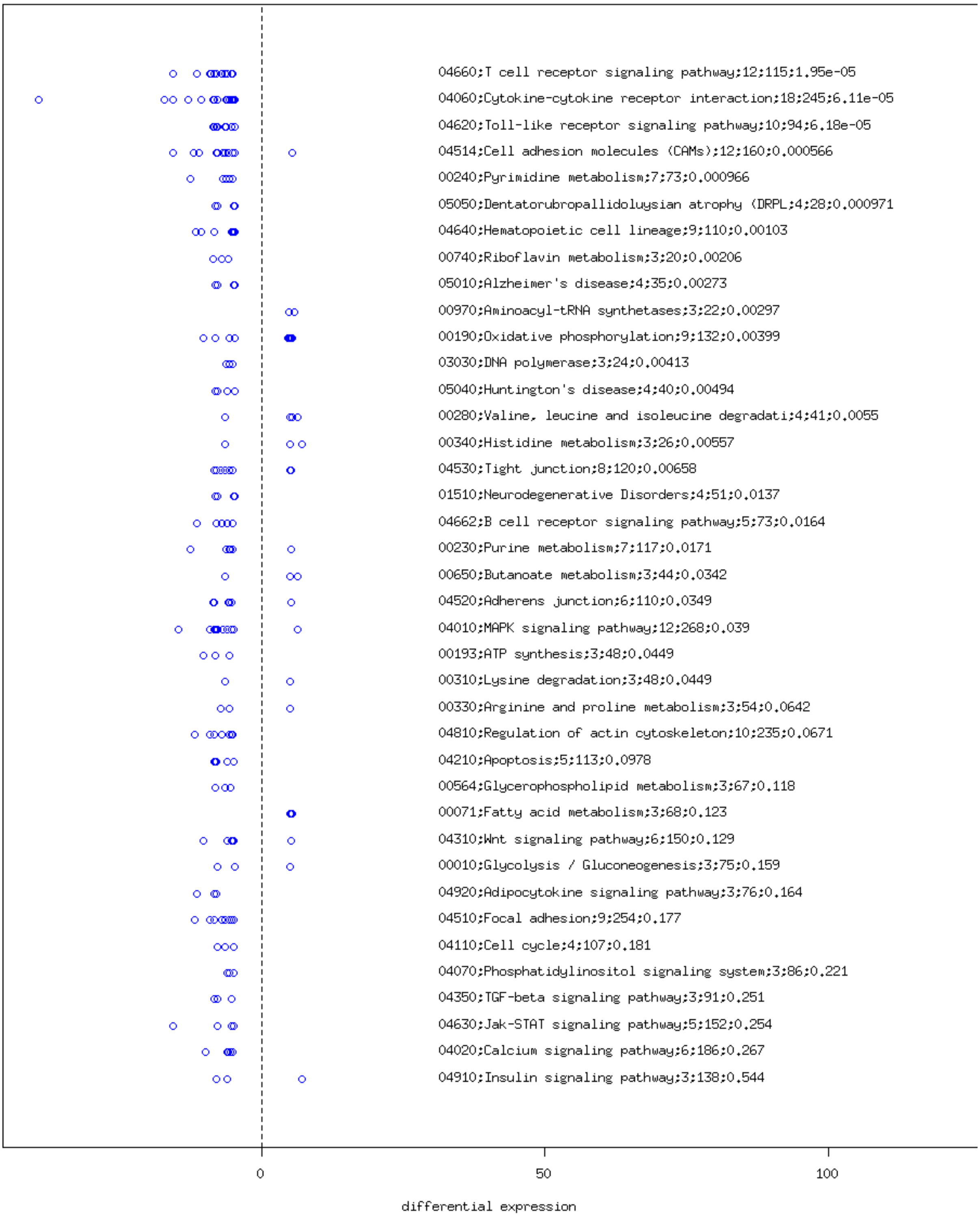

Figure 13: KEGG annotation terms for the experimental condition 8hpro-4hpro.

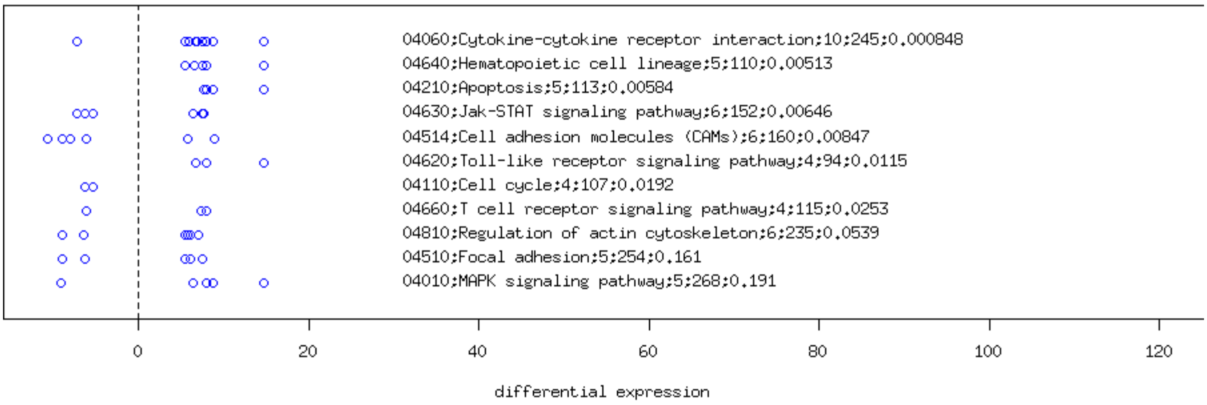

Figure 14: KEGG annotation terms for the experimental condition 4hko-0hko.

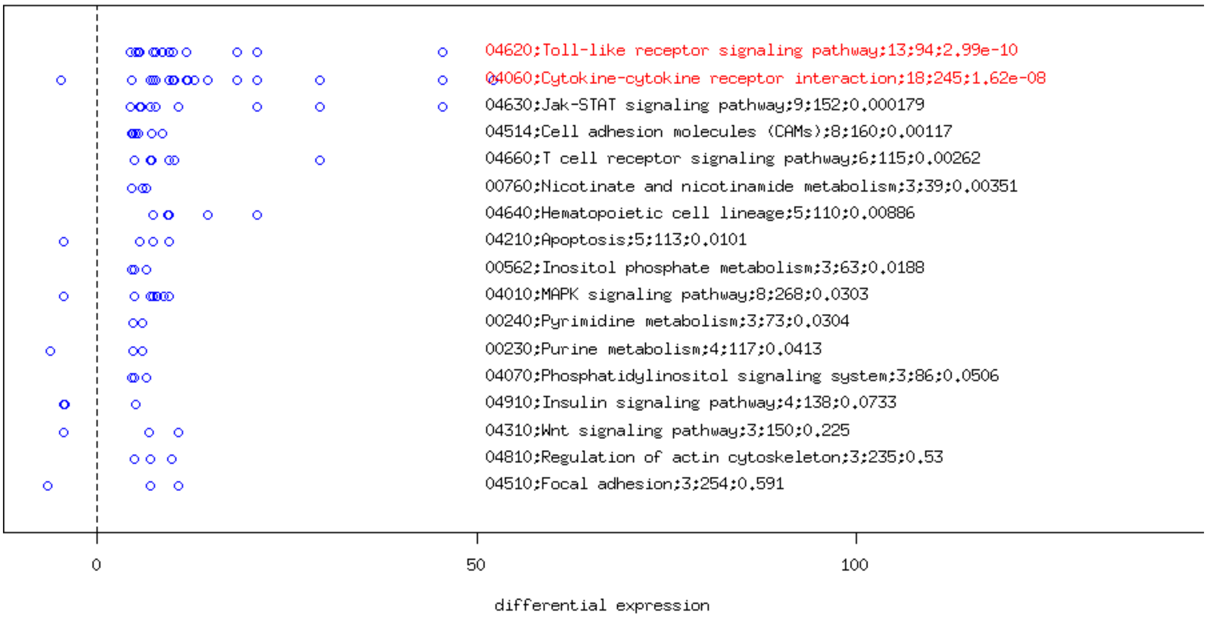

Figure 15: KEGG annotation terms for the experimental condition 4hko-4hpro.

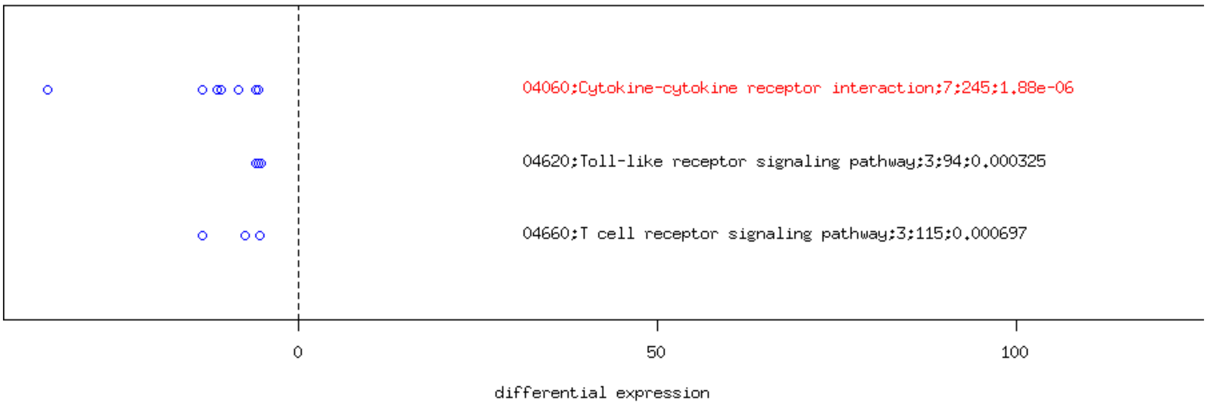

Figure 16: KEGG annotation terms for the experimental condition 8hko-8hpro.

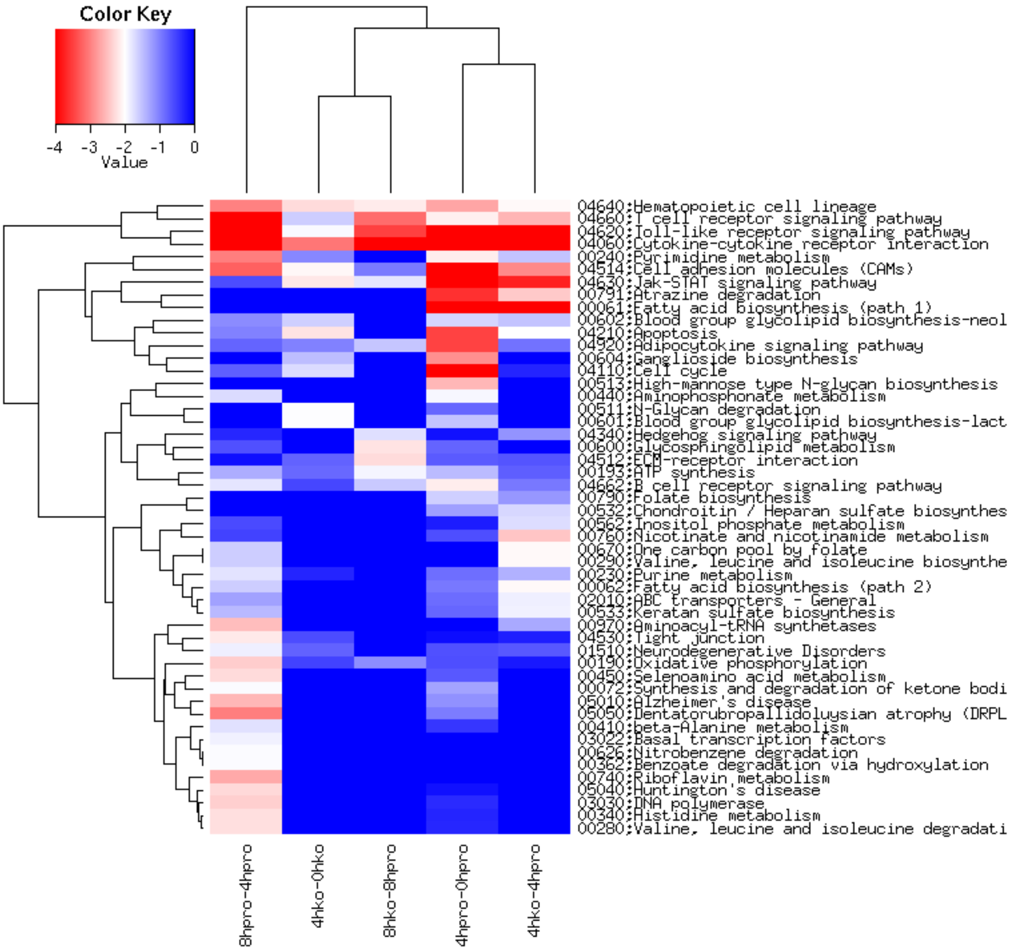

Figure 17: KEGG functional summary

5.2 GeneOntology annotation terms

GeneOntology (GO) provides three structured, controlled vocabularies (ontologies) that describe gene products in terms of their associated biological processes, cellular components and molecular functions in a species-independent manner. Given the GO data structure it is possible that different GO terms functionally characterize the same or nearly the same set of probesets. This is not an artifact and it is due to the fact that the selected probeset list map on the GO tree with terms that are close to each other (i.e. parents or children). Anyway an attempt of reducing this redundancy is made using the GOselection function provided in the AMDA library. This reduction of redundancy can be controlled starting AMDA via the advanced widgets interface and can be also excluded.

Figures from 18 to 22. If at least two experimental conditions are given a functional summary is provided (23) reporting the enrichment pvalues (log10 of pValues) for the different annotation terms and their resulting similarity on both direction of the heatmap.

In the currently used database built of Bioconductor annotation package (GO library, version 1.10.0) several thousands of GO terms are considered. Only GO terms represented with at least 3 DEG are ranked according to their relevance. Based on this ranking only the first 40 (or less whenever this number is not reached) are reported for plotting reasons.

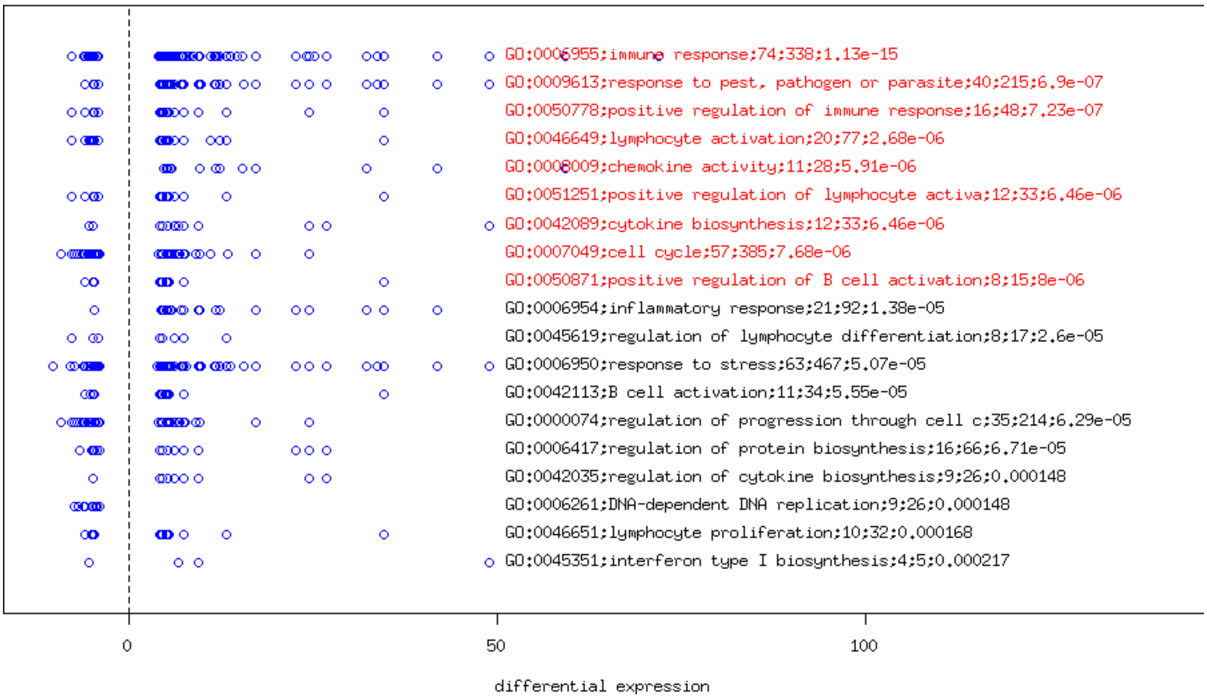

Figure 18: GO annotation terms for the experimental condition 4hpro-0hpro.

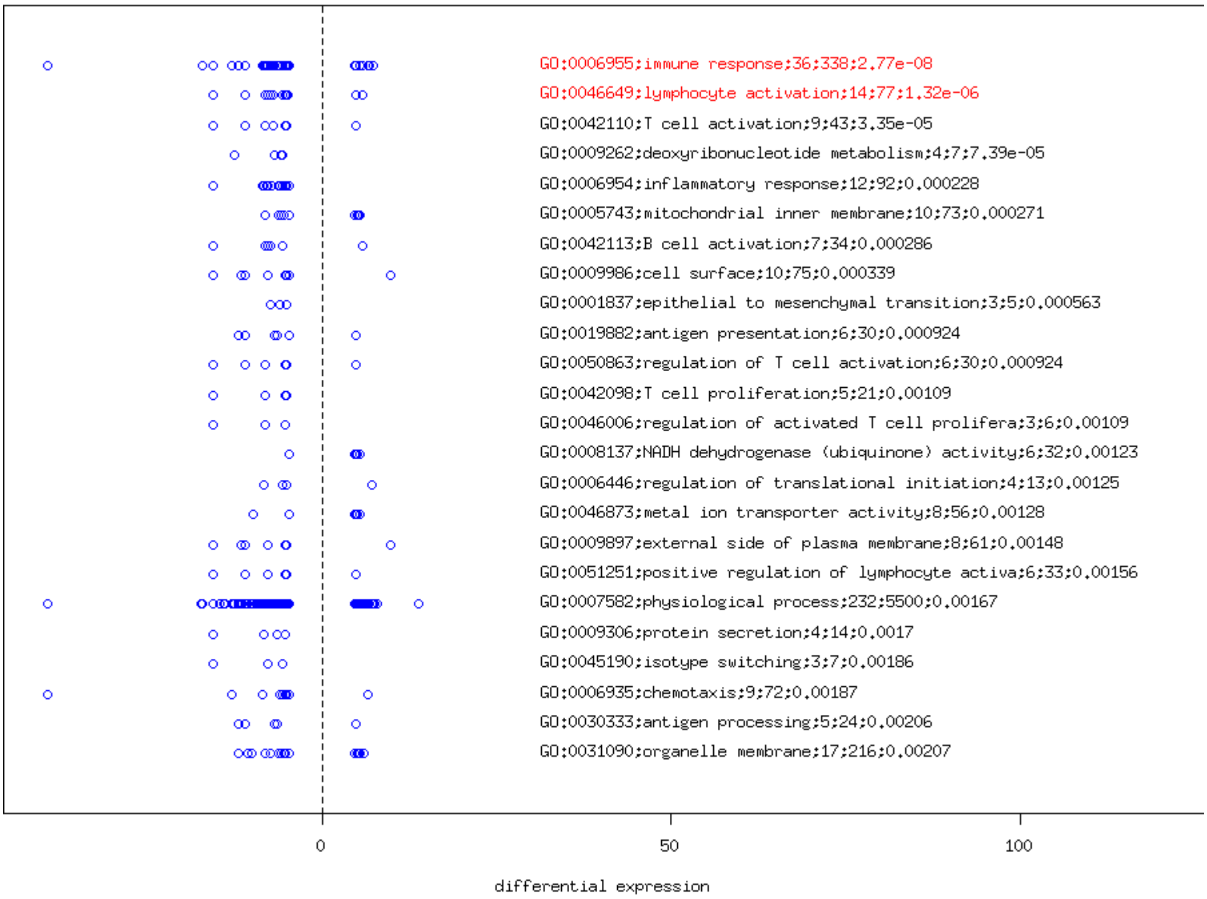

Figure 19: GO annotation terms for the experimental condition 8hpro-4hpro.

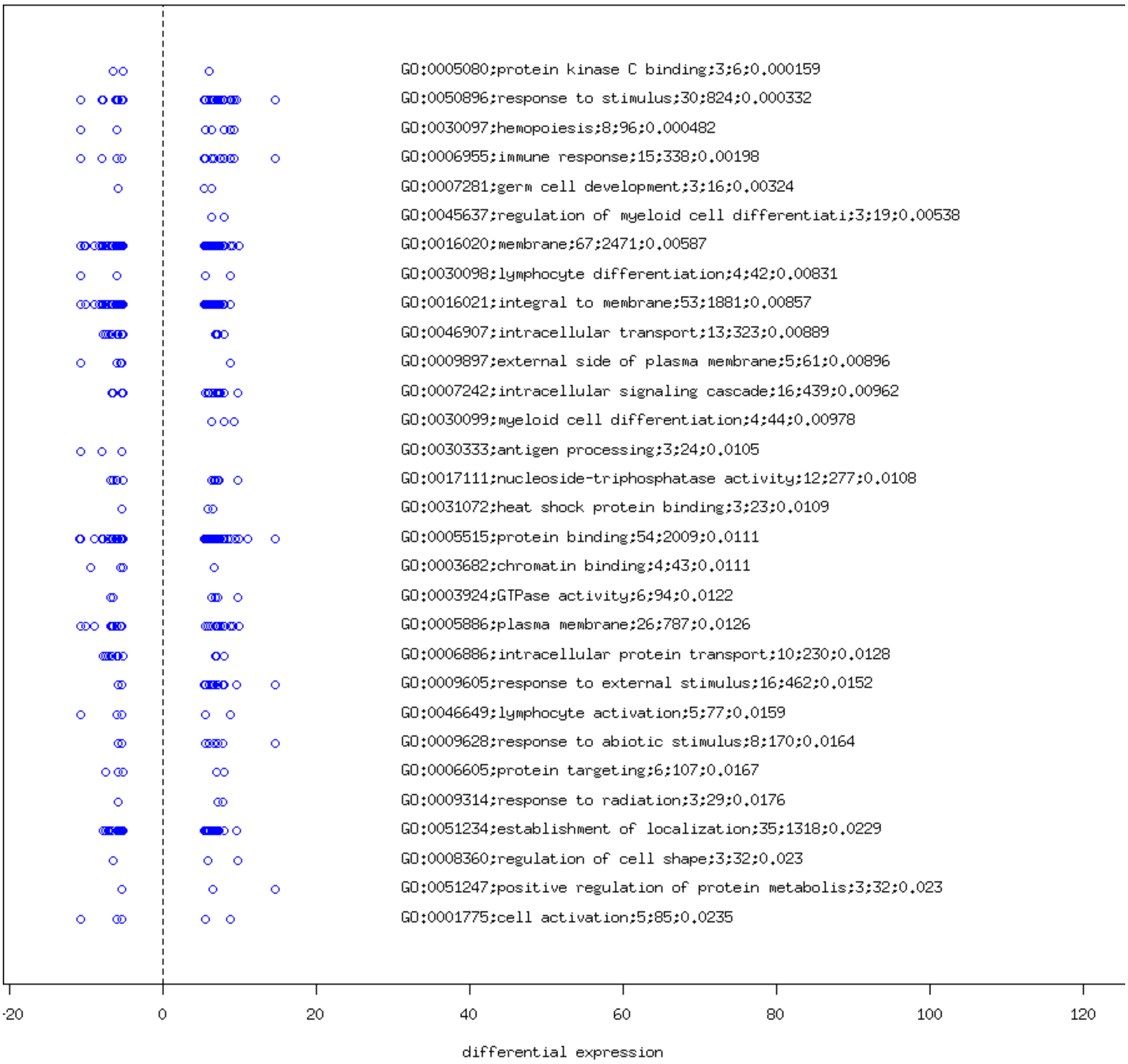

Figure 20: GO annotation terms for the experimental condition 4hko-0hko.

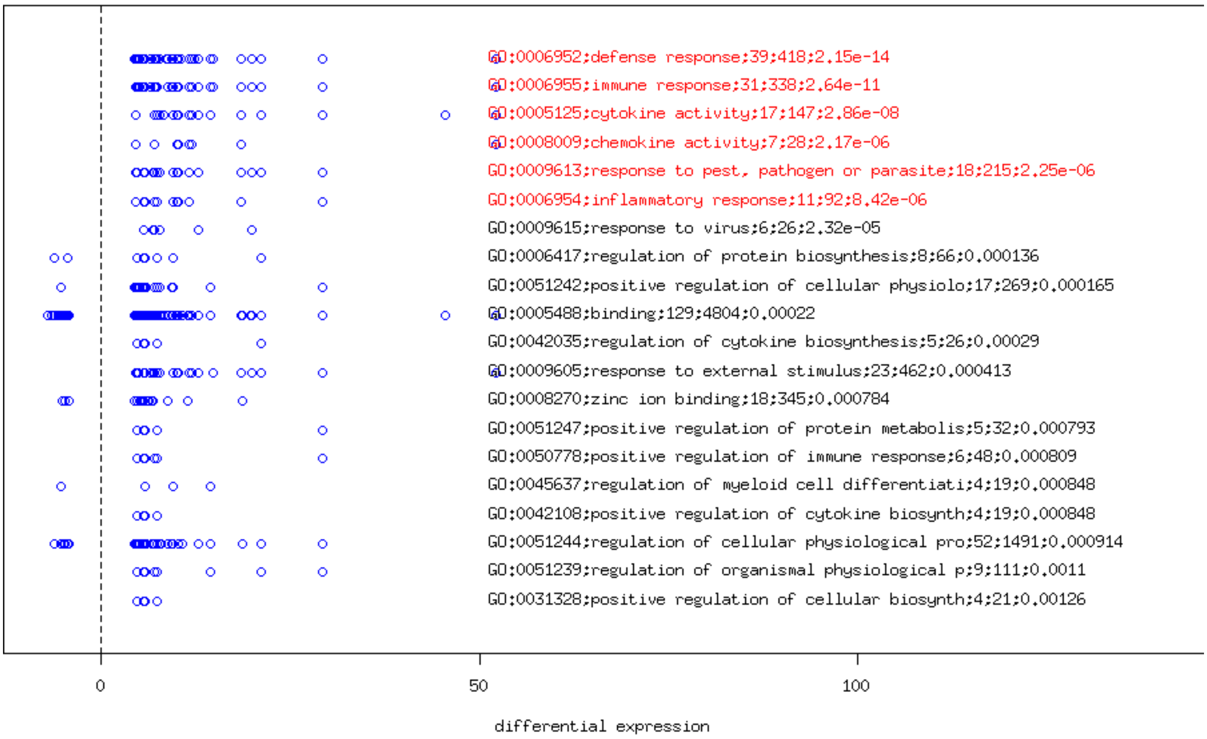

Figure 21: GO annotation terms for the experimental condition 4hko-4hpro.

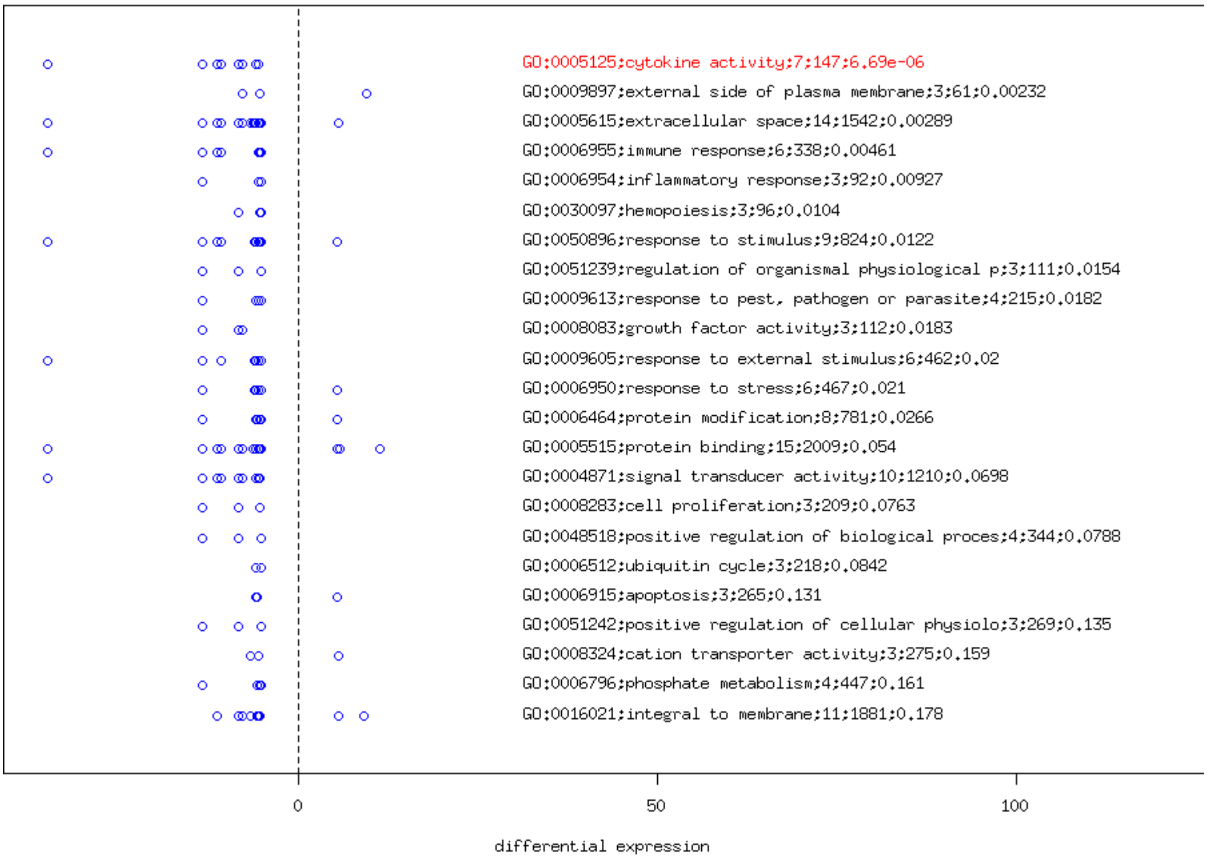

Figure 22: GO annotation terms for the experimental condition 8hko-8hpro.

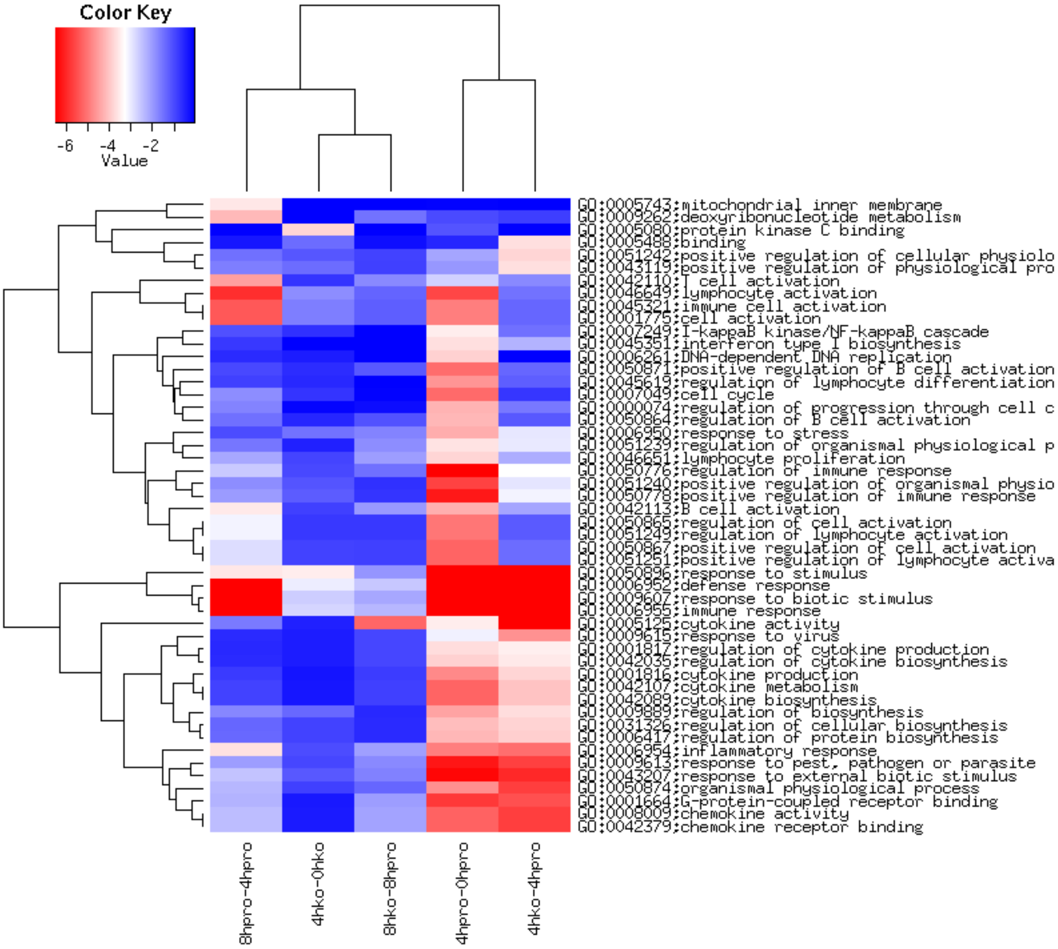

Figure 23: GO functional summary

5.3 User-provided annotation terms

User defined functional families are used to functionally characterize DEG of each experimental condition. This evaluation should provide to the user the information about the involvement of a priori expected functional families. Functional families are not filtered but only ranked based on their statistical relevance. Figures (from 24 to 28) are provided only for those functional families for which at least one DEG was found in the considered experimental condition. The observation could either confirm the expected involvement or provide unexpected insights (for example a gene family could be up-regulated when down-regulation was expected). If at least two experimental conditions are provided a functional summary is provided (29) reporting the enrichment pvalues (log10 of pValue) for the different annotation terms and their similarity.

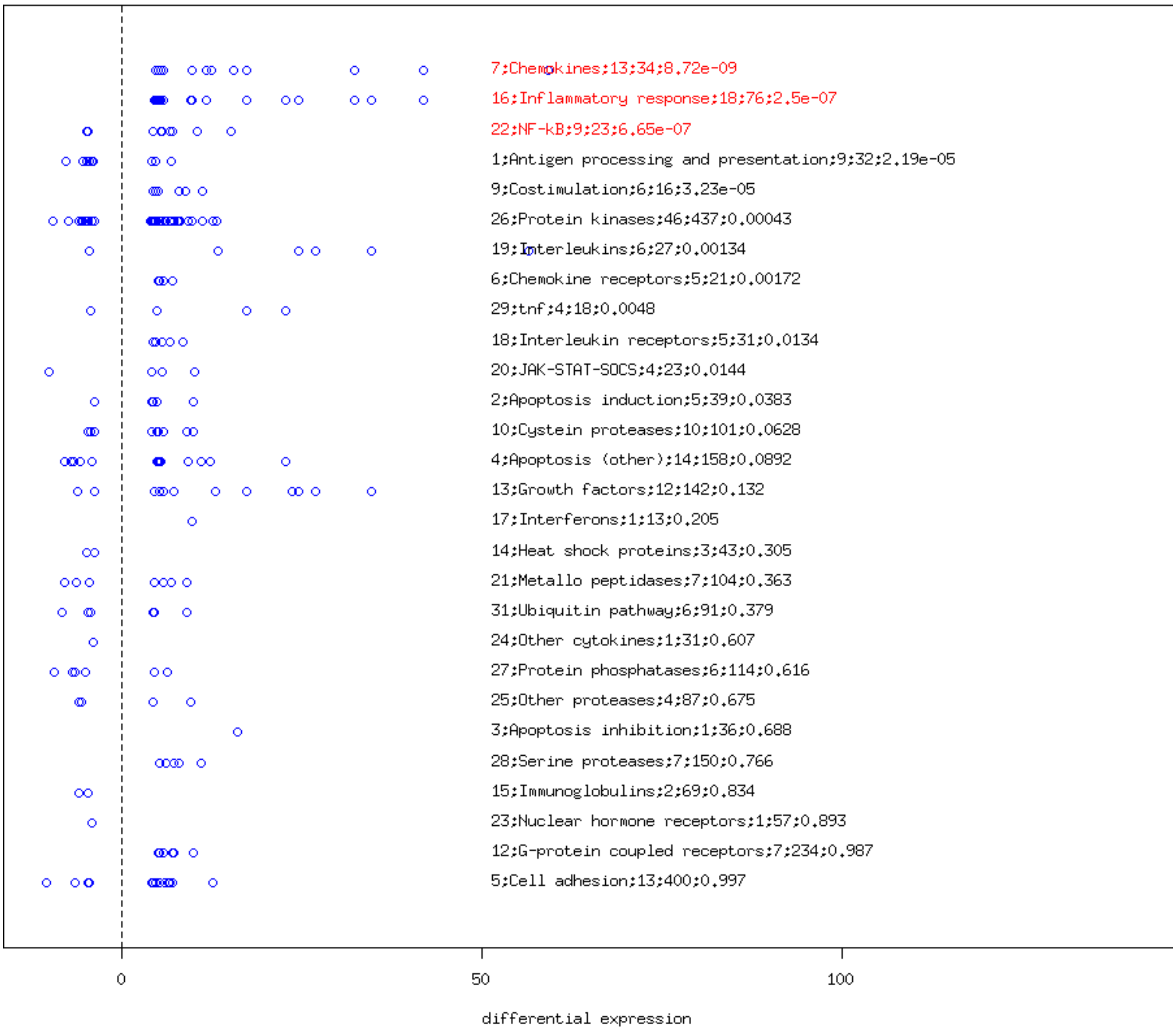

Figure 24: USER annotation terms for the experimental condition 4hpro-0hpro.

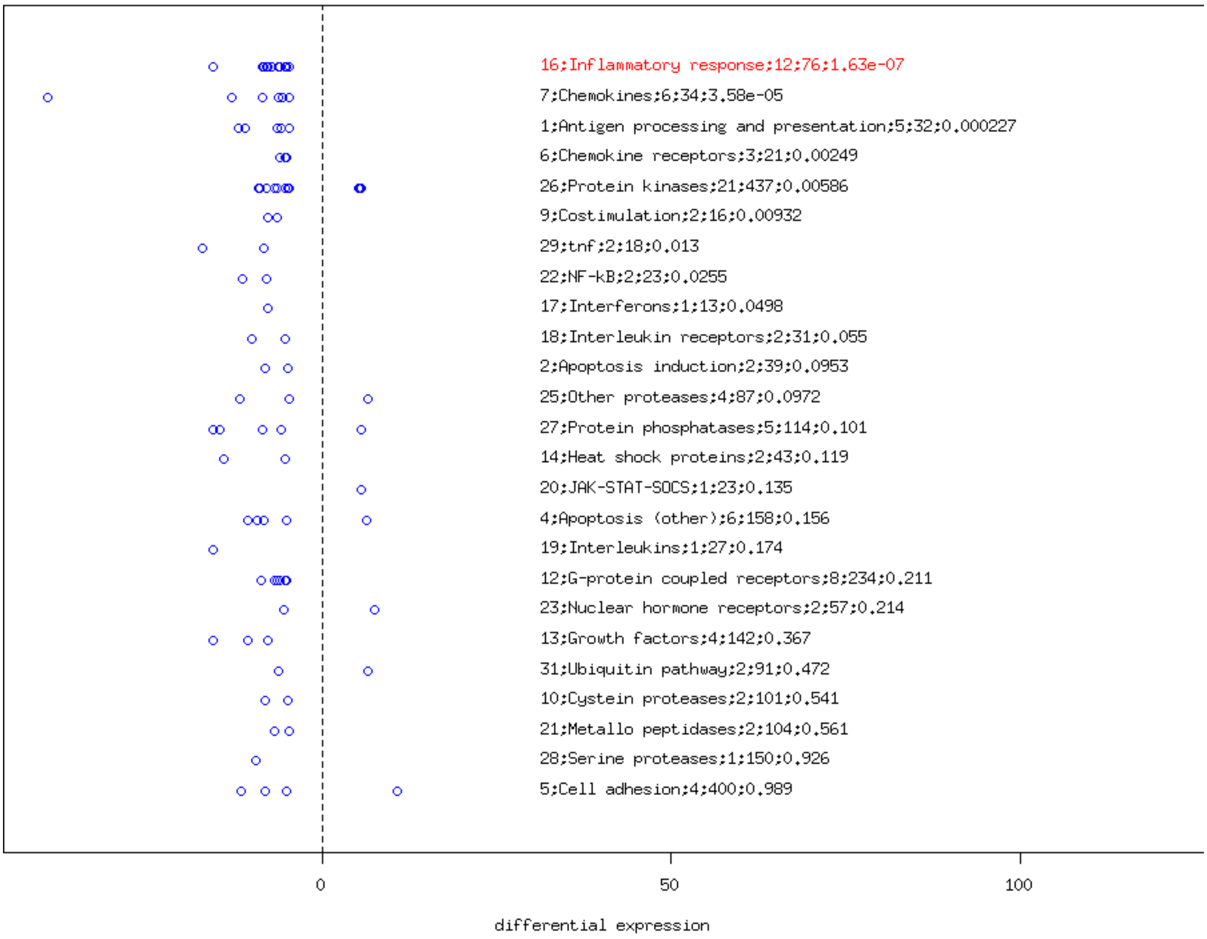

Figure 25: USER annotation terms for the experimental condition 8hpro-4hpro.

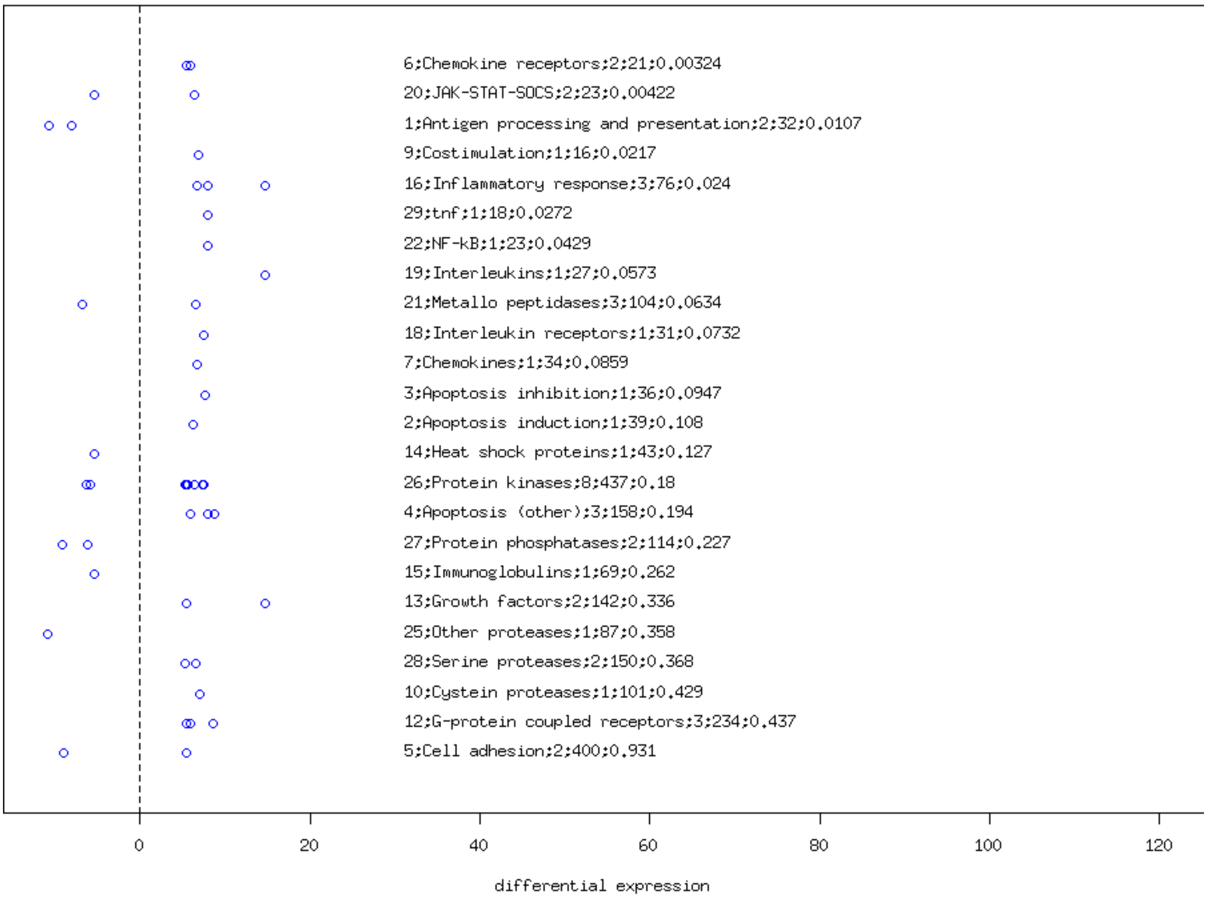

Figure 26: USER annotation terms for the experimental condition 4hko-0hko.

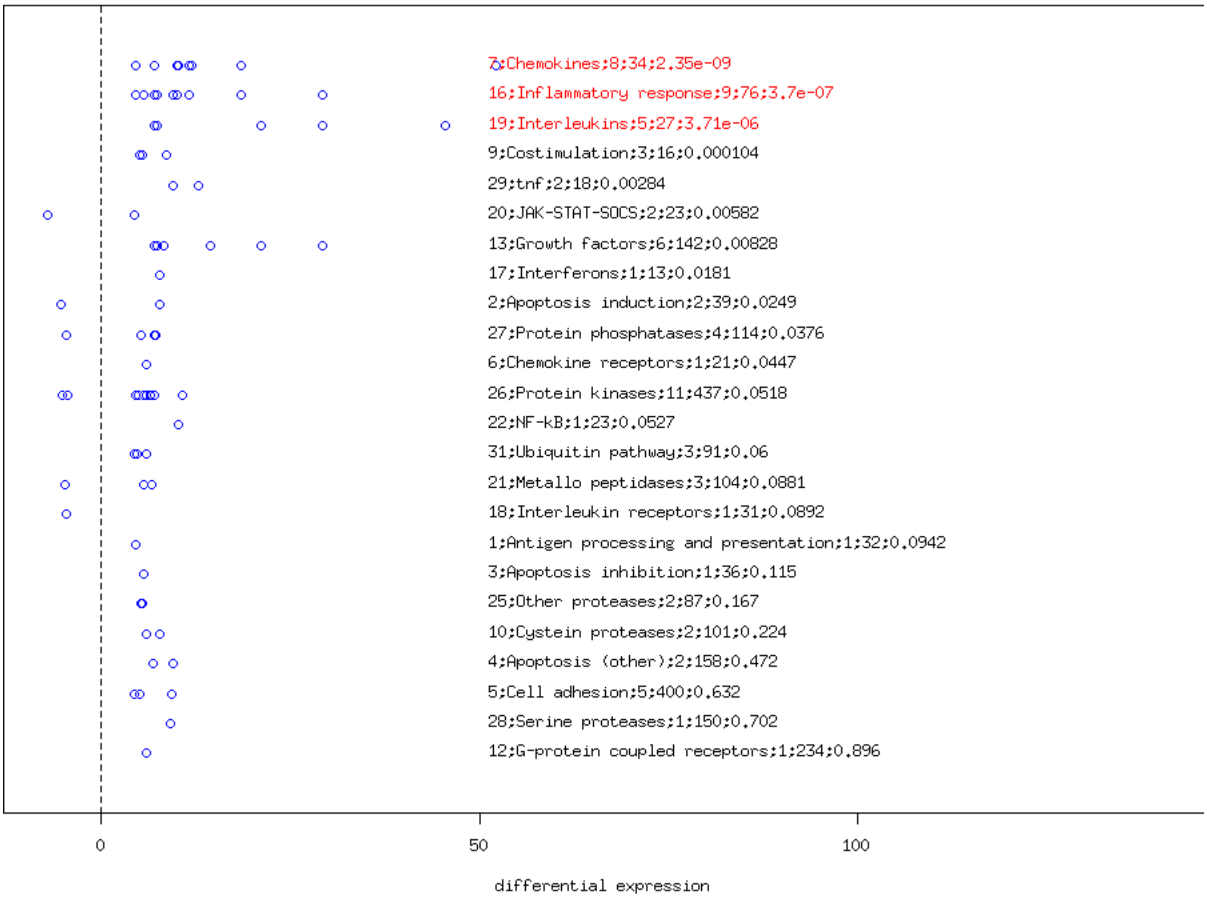

Figure 27: USER annotation terms for the experimental condition 4hko-4hpro.

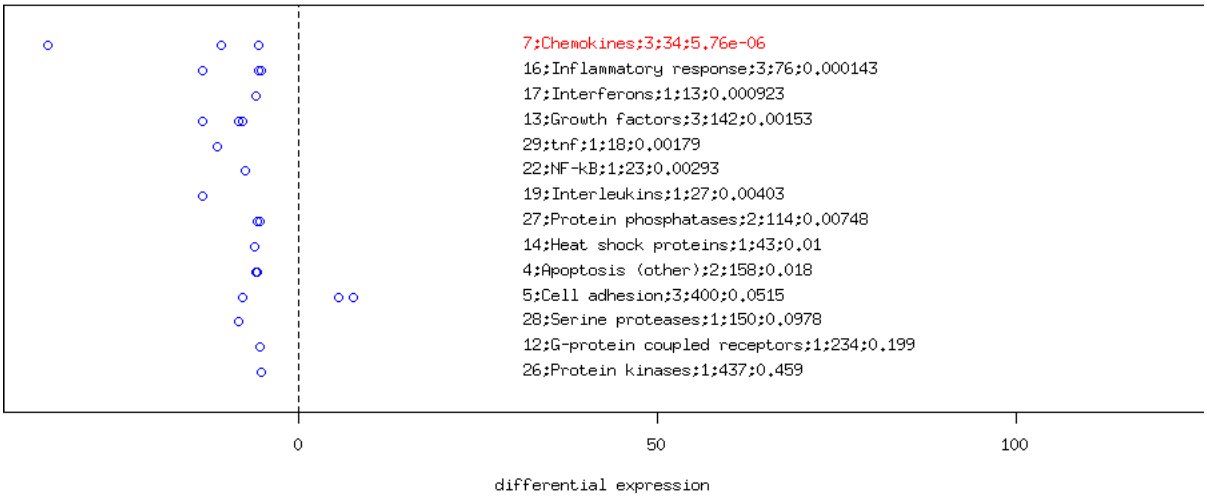

Figure 28: USER annotation terms for the experimental condition 8hko-8hpro.

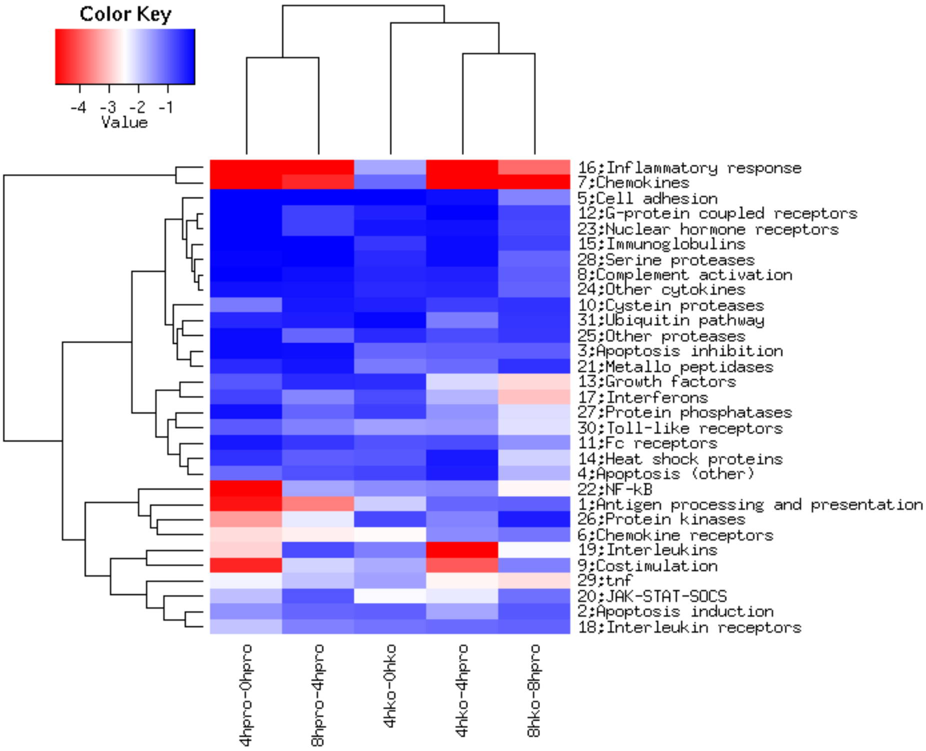

Figure 29: USER functional summary

6 Correspondence analysis

If a sufficient number of experimental conditions is provided (at least 3 conditions) a correspondence analysis is performed. This multivariate analysis technique aims to reveal relationships between genes and samples (Fellenberg et al., PNAS 2001).

Genes could be plotted in a n-multidimensional space (n the number of conditions or arrays), as well as contions or arrays could be plotted in a m-multidimensional space (m the number of genes). These multidimesional spaces can't obviously be graphically represented. Roughly, this technique allows for the reduction of the dimensionality of these spaces. In this way samples and genes can be represented in one bi-dimensional space.

The figure 30 represents the scores of arrays and DEG in the space of the two first "principal components". These new dimensions are the two directions in the original multidimensional space with the highest variance, and the scores can be imagined as the coordinates on these axis. Closeness of array labels in this space denotes similarity (like the hierarchical clustering of arrays). Each number represents an individual probeset detected as DEG in at least one experimental condition. Numbering follows the same ranking as in the DEG.universe html or txt file. Interesting genes can be identified also using the accessory function geneFilterCA provided in the AMDA library. The numbers close to the group of the interesting array labels are likely to be the most important in determining the similarity between those arrays.

It is possible to select the more representative genes that determine the specific features of different conditions. The annotation derived from these characteristic genes could give insights in to specific functional behavior of the corresponding samples.

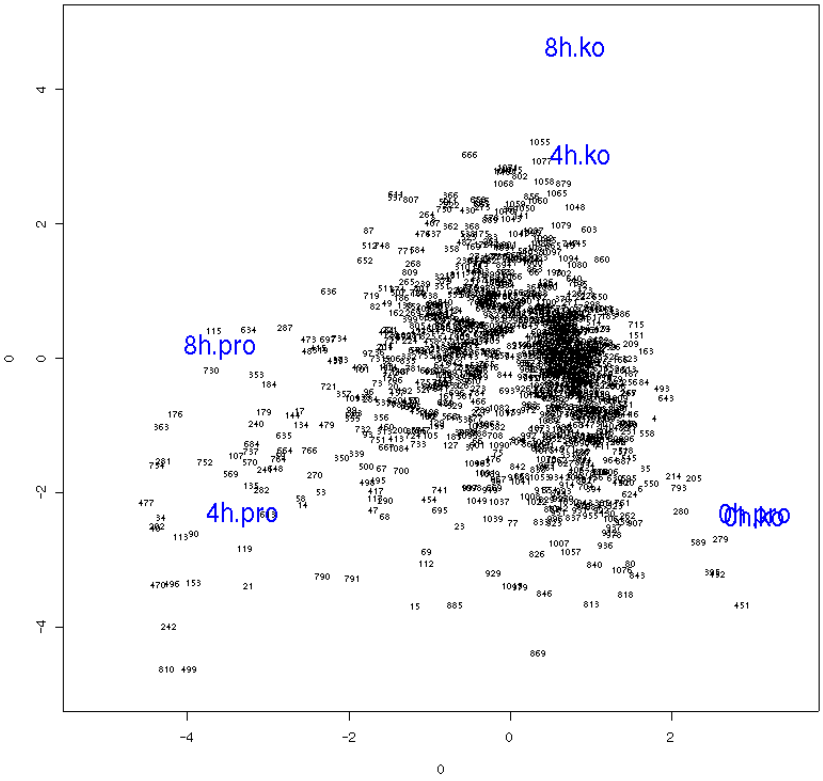

Figure 30: Correspondence analysis
